# Supplementary material for: HLA-Matched Allogeneic iPS Cells-Derived RPE Transplantation for Macular Degeneration
Source: J Clin Med. 2020 Jul 13;9(7):2217. doi: 10.3390/jcm9072217 (PMC7408794; doi:10.3390/jcm9072217)
Supplement: Supplementary file 1 [file jcm-09-02217-s001.pdf]

Supplementary Material

HLA-matched allogeneic iPS cells-derived RPE transplantation for macular degeneration

Sugita S, et al. *Corresponding author: E-mail: retinalab@ml.riken.jp

Material and Methods

Inclusion and exclusion criteria in age-related macular degeneration (AMD) patients

Table S1 presents the inclusion and exclusion criteria. Briefly, patients with advanced wet AMD in whom anti-VEGF treatment was not effective were included, while patients with any other significant ophthalmologic disease or previous history of malignancy except carcinoma in situ during the last 3 years were excluded. Candidate patients were scored for the existence of subretinal and intraretinal fluid, location and size of the subretinal lesion, and dominant eye and age. After obtaining informed consent, five patients with a high score from two study sites were enrolled. All clinical procedures were performed based on the clinical study protocols. All patients underwent systemic examination including a thorough cancer screening before and at 12 months after the transplantation surgery. The test results of the systemic cancer screening were evaluated by an independent board that consisted of medical oncologists. In one of five patients (Case 4), gastric carcinoma in situ was detected and endoscopic resection was performed prior to the transplantation surgery. No malignancies were detected in the other four cases before the transplantation surgery, and none of the patients were found to have any malignancy at 12 months after the transplantation surgery. Patients were followed up with periodical examinations. In brief, regular ophthalmological examinations including best-corrected visual acuity (BCVA), intraocular pressure measurement with Goldmann tonometry, slit lamp examination, funduscopic examination and spectral-domain optical coherence tomography (SD-OCT) scans were done at every visit. Fluorescein angiography (FA) and indocyanine green angiography (IA), multi-focal electroretinogram (ERG) using LE-4100 (Tomey, Nagoya, Japan), and microperimetry using MP-3 (Nidek, Gamagori, Japan) were carried out before surgery and at 3, 6 and 12 months after surgery. The National Eye Institute Visual Functioning Questionnaire 25 (VFQ-25) was administered before and at 1, 3, 6 and 12 months after surgery.

Genome and methylation analyses for iPS cells (iPSC) and RPE cells

We established iPSC from a HLA homozygote healthy donor. Genomic mutation analyses were performed with peripheral blood mononuclear cells of the donor (termed origin), one iPSC line (n=3), QHJI01s04, and two RPE samples that were both differentiated from QHJI01s04 cells. The number of genomic mutations and the sequencing statistics are summarized in **Tables S2** and **S5**, respectively. **Tables S3** and **S4** show the details of the mutations**.** For the methylation analysis, we additionally used data from 56 samples for comparison, with 53 selected from normal tissues from GSE31848 (https://www.ncbi.nlm.nih.gov/geo/query/acc.cgi?acc=GSE31848), two from human eye samples, and one RPE sample purchased from LONZA. The resultant methylation pattern is shown in **Fig. 1**. **Table S6** shows the primers used in Amplicon sequencing to validate the SNVs found in the iPSC and RPE cells.

SNP-genotyping array

CNV analysis was performed using HumanOmniExpress-24 v1.1 (Illumina). We labeled and hybridized genomic DNA (200 ng) on a DNA Beads chip. We generated a final report and performed the cnvPartition CNV analysis (v3.2.0) using GenomeStudio (2011.1) (Illumina). The CNVs were determined using PennCNV [1] (1.0.3), Mosaic Alteration Detection-MAD [2] (1.0.1) and GWAS tools (1.16.1). After comparing the results between the origin and the samples, we were able to extract the CNVs from only the samples.

Whole genome and whole exome sequencing

Whole genome sequencing (WGS) libraries were generated using KAPA Hyper Prep Kit (Roche) or Nextera DNA (Illumina) from 200 ng and 50 ng of genomic DNA, respectively. Whole exome sequencing (WES) libraries were generated using Nimblegen SeqCap EZ Library v3 (Roche) from 1 µg of genomic DNA. Libraries were sequenced on HiSeq2500 (Illumina) using HiSeq SBS Kit v4. All experiments were performed according to the manufacturer's instructions. After generating FASTQ files with Bcl2fastq (1.8.4), trimming adapter sequences with Trim Galore (0.4.1) and performing a quality filter with Qcleaner (3.1) (Amelieff) in the case of KAPA Hyper Prep Kit, sequencing reads were aligned to the human genome reference (hg19) including the decoy (hs37d5), the plasmid (pCE-hOCT4, pCE-mp53DD, pCE-hSK, pCE-hUL and pCXB-EBNA1) and PhiX sequences using Burrows-Wheeler Aligner (BWA-MEM, 0.7.10 r876) (http://bio-bwa.sourceforge.net/). The duplicate reads were removed using Novosort (1.03.01) (Novocraft Technologies).

Mutation calling and annotation

Mutation (SNV and insertion/deletion) calling on paired samples was performed using Genomon-exome (1.0.1) (http://genomon.hgc.jp/exome/) with Fisher's exact test (P<0.001, strand ratio ≠0 or 1 variant allele frequency in the founder cells < 0.1) and Genomon (2.0.5) (https://github.com/Genomon-Project) with EB call (Fisher (P-value >=1.0, EBCall (P-Value) >=3 for WGS, EBCall (P-Value) >=4 for WES, variantPairNum_tumor >=4, P-value (Fisher)_realignment >=1.0, strand ratio ≠0 or 1). Mutations that satisfy the following conditions were considered: the ratio of sample alternative allele frequency and control alternative allele frequency >=5 and sample alternative allele frequency >= 0.05. Functional annotation was performed using ANNOVAR 2013 July 28 followed by annotation filters as follows: In the first step, we retained mutations on CDS and splicing regions and excluded synonymous SNVs. Secondly, we then excluded mutations listed in dbSNP 131, ESP 6500 (>0.01), 1000g2014oct (>0.01) and Human Genetic Variation Database (HGVD) (>0.01), if they were not listed in the Catalogue of Somatic Mutations in Cancer (COSMIC) version 74, Cancer Gene Census, Shibata's list (http://www.pmda.go.jp/files/000152599.pdf#page=8) and the Human Gene Mutation Database (HGMD). The DNA copy number analysis with paired samples was performed using WGS data. CNVs were called using VarScan [3] 2.3.7 (log2_ratio < -0.415 or log2_ratio > 0.322 or Otsu's threshold [4]) and Delly [5] 0.5.6. (SVLEN > 1500 and Filter == "PASS" and sample|FT == "PASS" and control|FT == "PASS"). Finally, we manually excluded suspicious CNV candidates.

Amplicon sequencing

We designed the primer using primer.py (Amelieff) or the Primer3Plus website (http://primer3plus.com/cgi-bin/dev/primer3plus.cgi) and Primer-BLAST website (https://www.ncbi.nlm.nih.gov/tools/primer-blast/). For the multiplex PCR reaction, we mixed 10 ng of genomic DNA, 200 mM of the primers in the **Table S6** and KAPA HiFi HotStart ReadyMix (Roche) with 5% DMSO. The PCR condition was as follows: the initial denaturation was done at 95°C for 5 min, followed by 35 cycles of denaturation done at 98°C for 20 sec, annealing at 60°C for 15 sec, extension at 72°C for 30 sec and final extension at 72°C for 1 min. We purified the amplicon using the 1.8×Agencourt AMPure XP Beads (Beckman Coulter). Subsequently we then generated the sequencing library using KAPA Hyper Prep Kit for Illumina (Roche). The library was sequenced on MiSeq (Illumina) using MiSeq Reagents Kit v2 according to the manufacturer's instructions. The sequencing reads were aligned to the reference genome used in WGS/WES with the alternative allele frequencies at the targets then calculated.

DNA methylation array

Bisulfite conversion of 500 ng genomic DNA was performed using EZ DNA Methylation Kit (Zymo Research). DNA methylation profiling of the bisulfite-converted DNA was performed using HumanMethylation450K BeadChip (Illumina) according to the manufacturer's instructions. After exporting methylation data using GenomeStudio (2011.1) and normalizing beta values using BMIQ,[6] we averaged the beta values of 74 genomic blocks at the transcription start sites of cancer related genes as previously described [7], with visualization performed using R (https://www.R-project.org/).

Preparation of iPS-RPE cells and cell quality assessment

The differentiation of iPS cells into RPE cells has been previously described [8]. In the present study, we tested 4 iPS-RPE cell lines for the quality standard and assessment (**Tables S8** and **S9**). The quality of the iPS-RPE cells (morphology, cell viability, RPE-related genes (RPE marker), RPE purity) was assessed according to the list of requirement standards used in our previous report [8] with minor modification. The contamination of undifferentiated cells (Lin28 positive) was evaluated by qRT-PCR. Endotoxin was checked according to protocols in the Japanese pharmacopoeia. The secretion of PEDF and VEGF by iPS-RPE cells was measured in culture supernatants using PEDF ELISA kit (BioVendor) and VEGF-A ELISA kit (eBioscience) [8].The specific details have been described in our previous study [8].

Phagocytosis of shed photoreceptor rod outer segments

To confirm phagocytic function, iPS-RPE cells (QHJI01s04 line) were cultured with RPE medium in the presence of FITC-labeled porcine shed photoreceptor rod outer segments (ROS, 10 μg/cm^2) for 8 hr at 37°C or 4°C as a control. Untreated RPE cells without ROS were also prepared as the control cells. After incubation with FITC-ROS, RPE cells were treated with 0.25% trypsin-EDTA, and phagocytosis was evaluated by microscopy. The specific details have been previously described [8].

Tumorigenicity test

Using immunodeficient mice, iPS-RPE cells (QHJI01s04 line) were tested for tumorigenicity. This test involved the subcutaneous injection of RPE cells with 200 μL Matrigel (BD Biosciences) in NOG mice (CLEA Japan, Inc.). We subcutaneously injected iPS-RPE cells (1×10^6 cells) or HeLa cells (1×10^6 cells, as a positive control) with Matrigel in NOG mice. If the mice survived and had no palpable tumors, they were sacrificed and used to measure the sizes of the transplants at 12 weeks (n=2), 24 weeks (n=1), 42 weeks (n=1), and lifelong (65 weeks, n=2). Matrigel without cells was injected as a negative control. When the size of the transplant was almost the same as that of the Matrigel only vehicle, the transplant was judged as negative for tumorigenic potential. The specific details have been described in our previous study [8].

PCR test for infectious pathogens

To examine infectious pathogens in RPE cell cultures, we performed PCR in the RPE cells. After collecting samples, DNA and RNA were extracted from RPE cells (QHJI01s04 line) (n=2). As per the details in previous reports,[9, 10] multiplex real-time PCR was conducted for the purpose of detecting the following pathogens: herpes simplex virus type 1 (HSV-1), type 2 (HSV-2), varicella-zoster virus (VZV), Epstein-Barr virus (EBV), cytomegalovirus (CMV), human herpes viruses type 6 (HHV-6), type 7 (HHV-7), type 8 (HHV-8), parvovirus B19, BK virus (BKV), JC virus (JCV), hepatitis B virus (HBV), hepatitis C virus (HCV), human immunodeficiency virus type

1 (HIV-1), type 2 (HIV-2), human T-lymphotrophic virus type 1 (HTLV-1), and type 2 (HTLV-2). PCR was performed with a LightCycler 480 II instrument (Roche, Basel, Switzerland). In addition, we also prepared positive and negative control cells for this PCR. The specific details have been described in our previous study [8].

Mycoplasma tests

In our clinical study of iPS-RPE transplantation, mycoplasma was checked according to the protocols presented in the Japanese pharmacopoeia (mycoplasma sterility tests) [8]. Moreover, in addition to the above tests, we also conducted a novel mycoplasma PCR test (Myco Finder, Nissui Pharmaceutical Co., Ltd.) using transplanted iPS-RPE cells on the day of surgery. As a result, this infection was checked twice in the present clinical study.

Expression of HLA molecules on iPS-RPE cells

To confirm the expression of Human Leukocyte Antigen (HLA) molecules on RPE cells, we examined the expression of HLA-class I (A, B, C) and class II (DR, DQ, DP) on transplanted iPS-RPE cells. Before the assay, RPE cells were prepared with IFN-γ pre-treatment (human recombinant IFN-γ 100 ng/mL, 48 hr). Information for the antibody and the methods have been previously described.[11] The samples were analyzed using FACSCanto™ II or FACSAria™ II flow cytometer (BD Biosciences, San Jose, CA). Data were analyzed using FlowJo software (version 9.3.1).

Area measurement of window defect lesions in fluorescein angiography images

Fluorescein angiography (FAG) was performed at the Kobe City Eye Hospital and Kobe City Medical Center General Hospital according to the schedule for this study. The Heidelberg Spectralis HRA (Spectralis; Heidelberg Engineering, Heidelberg, Germany) was used to record FAG images at the angle of 25°, which was aimed at the macular region. The images used for window defect evaluation were selected from those obtained at less than one minute after initiation of the imaging. The window defect area was evaluated using the images that are described below.

Evaluation of binarized window defect lesions in the FAG images

Binarization of the window defect lesion in the FAG images was performed using an Otsu method. Briefly, the FAG image was analyzed by the Fiji software (ImageJ version1.52g, provided in the public domain by the National Institutes of Health, Bethesda, MD, USA; http://imagej.nih.gov/ij/). The selected image was converted to 8 bits. The background brightness of more than six regions were randomly selected by the Oval Selection Tool of the Fiji software sparing retinal veins in the vascular arcade area. The averaged brightness was set at the minimum value in order to minimize the noise in the FAG images. The threshold of each image was then adjusted using the Otsu method under a dark background, with the lesions representing retinal veins removed manually where possible. After the dark background setting was removed in order to convert the binarized image with a white background, the area was selected by the create selection setting from the edit tools so that the window defect area could be measured. This measurement was independently performed three times.

Evaluation of pigmentation in RPEs in the grafted area

A polarization-sensitive OCT (PS-OCT) has been employed in ophthalmic imaging as a way to measure the birefringence of fibrous tissues [12-14]. As polarization scrambling at the RPE via melanin granules has been observed [15, 16], this has been utilized for the segmentation of the RPE [17]. To specifically detect the melanin at the RPE layer in a subject in the present study, we used a clinical prototype of PS-OCT (Tomey Corporation, Nagoya, Aichi, Japan), which provided entropy of local Jones matrices as a measure of spatial randomness of the polarization property by Cloude-Pottier decomposition [18]. The light source was a frequency-swept laser (Axsun Technologies, MA, US) with a center wavelength of 1.05 μm, a wavelength range of 100 nm, and a wavelength scanning rate of 100 kHz. The interferometer of the PS-OCT was based on the depth-encoding method with a similar configuration previously demonstrated with the exception for the wavelength band [19] . The entropy is dimensionless and has a range from 0 (totally uniform) to 1 (totally random polarization property). A lateral range of 6 mm × 6 mm at the macular area was scanned with 1024 × 256 A-scans. Entropy was calculated with a kernel size of 11 (width) × 5 (depth) pixels, which corresponds to 64 μm (width) × 18.6 μm (depth) [18]. Bruch's membrane line was marked on each B-scan image, and the signal below the line, which corresponds to the choroidal area, was excluded from the analysis. Assuming that only the melanin pigment of RPE caused the polarization scrambling in the inner retina of the subject, the highest entropy in the inner retina above the Bruch's membrane was extracted for each A-scan and used to create the *en face* entropy map (**Figure S6A**), which reflected the melanin distribution of the RPE. The macular entropy was then calculated as a mean of the *en face* entropy map in a circular region centered at the fovea with a 3-mm diameter. Low entropy area was defined as a ratio of the area where the *en face* entropy is less than 0.2 to the total area in the same circular region (**Figure S6B-D**).

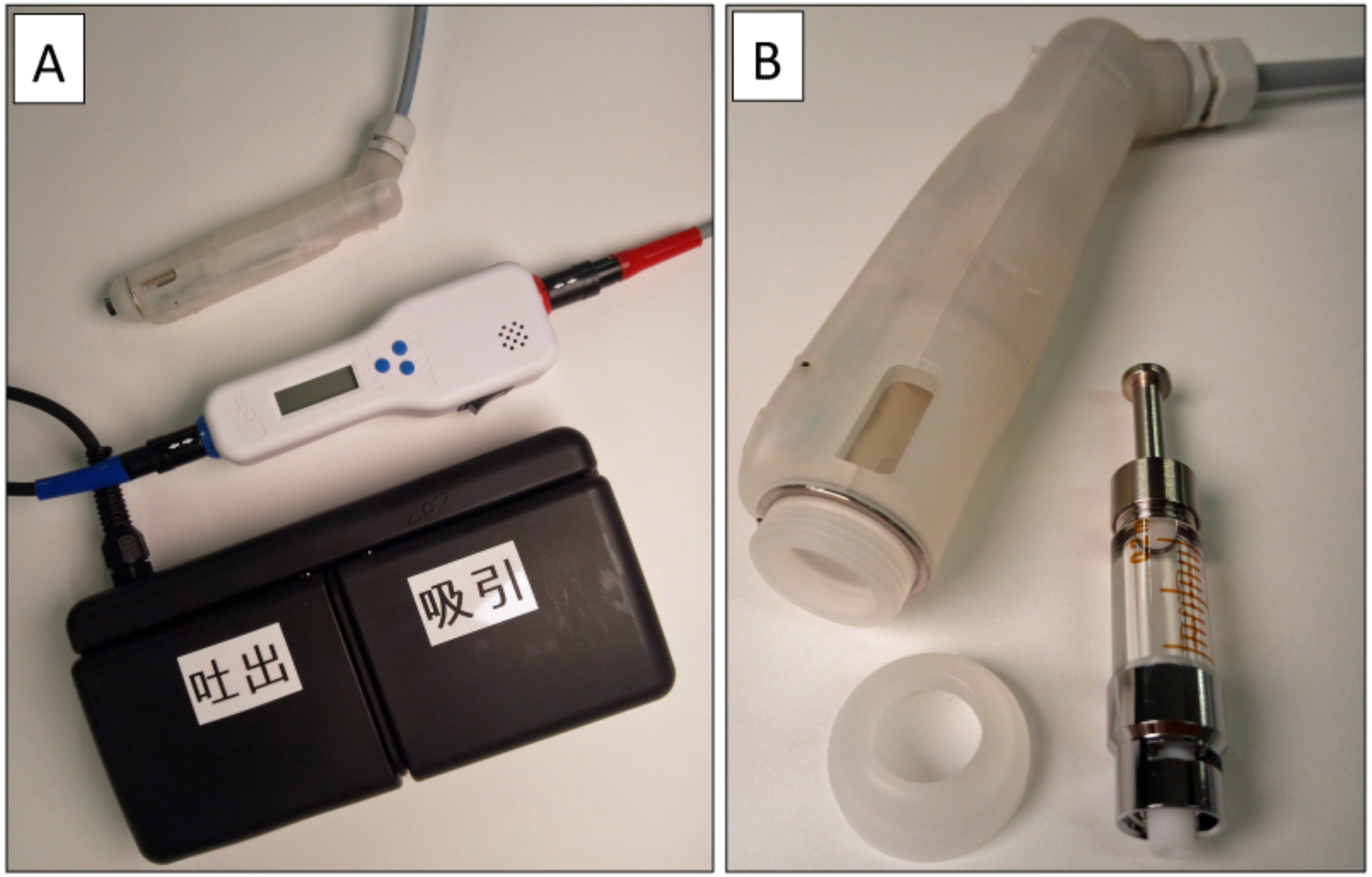

Fig. S1. Instrument for iPS-RPE transplantation surgery.

(**A**) The operator held the upper injector (WRRK-02; Icomes Lab) and placed the tip of the cannula (PolyTip® cannula 25g/38g; MedOne) into the subretinal space. The operator or assistant injected the RPE cell suspension by using the lower foot pedals. The operators were able to control injection speed and read the amount of fluid injected with the middle controller. (**B**) The cell suspension was loaded in the lower 0.2 mL syringe installed in the upper injector.

Clone	QHJI01s04-RPE-01						
Weeks	12w			24w	42w	65w	
Mouse ID	NOG230-1	NOG230-2	NOG229-3 (HeLa)	NOG231-1	NOG232-2	NOG232-1	NOG229-2 (matrigel)
graft	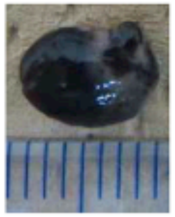	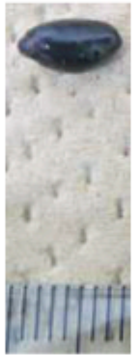	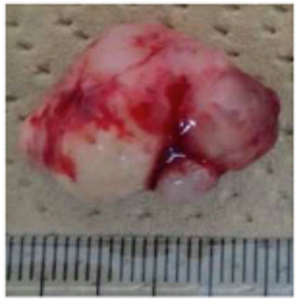	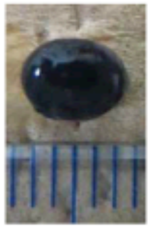	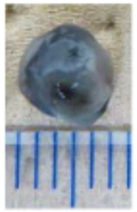	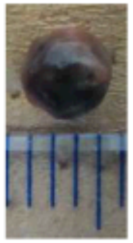	Not found
graft size D x W x H	7 mm x 7 mm x 3 mm	4 mm x 7 mm x 4 mm	1.4 cm x 2.3 cm x 1.0 cm	4 mm x 5 mm x 3 mm	4 mm x 5 mm x 3 mm	4 mm x 4 mm x 3 mm	

Fig. S2. Tumorigenicity tests for iPS-RPE cells.

Before transplantation, we used these iPS-RPE cells to test the tumorigenicity. Immunodeficient mice (NOG mice) were used to test the tumorigenic potential of the RPE cells. To assess the tumorigenicity of iPS-RPE cells (QHJI01s04 line), we conducted tests using subcutaneous transplantation of iPS-RPE cells (1×10^6) or HeLa cells (1×10^6) with 200 μL Matrigel. At 12 weeks, the size of the cell clumps from the iPS-RPE cells were small compared with positive control cells (HeLa cells), even when RPE cells were injected at 24, 42 or 65 weeks. No tumors were observed in these mice. HeLa cells developed a large mass by 12 weeks. D × W × H - Depth × Width × Height.

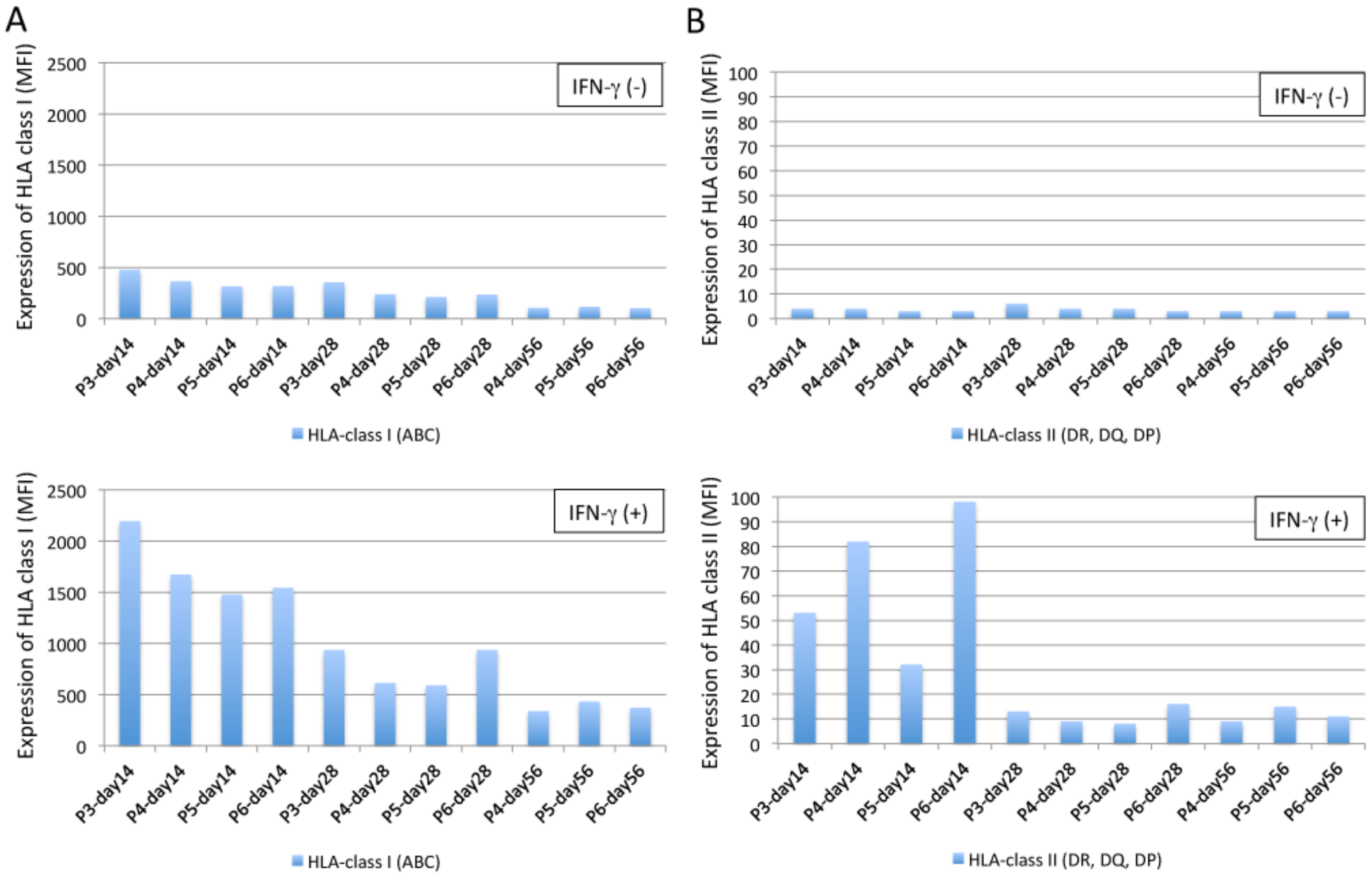

Fig. S3. Expression of HLA class I and class II on transplanted iPS-RPE cells by FACS analysis.

(**A**) iPS-RPE cells (QHJI01s04 line) constitutively expressed HLA class I (ABC), and RPE cells exposed to IFN-γ greatly expressed class I molecules. (**B**) Although the iPS-RPE cells did not express HLA class II (DR, DQ, DP), the RPE cells exposed to IFN-γ expressed class II molecules. During the cultures, P3-P6-day14 cultured cells highly expressed HLA class I and II under IFN-γ exposure. In contrast, P3-P6-day28 cells poorly expressed HLA class I and II. P3-day14 indicates 3 passages and a culture time for 14 days. MFI - mean fluorescence intensity.

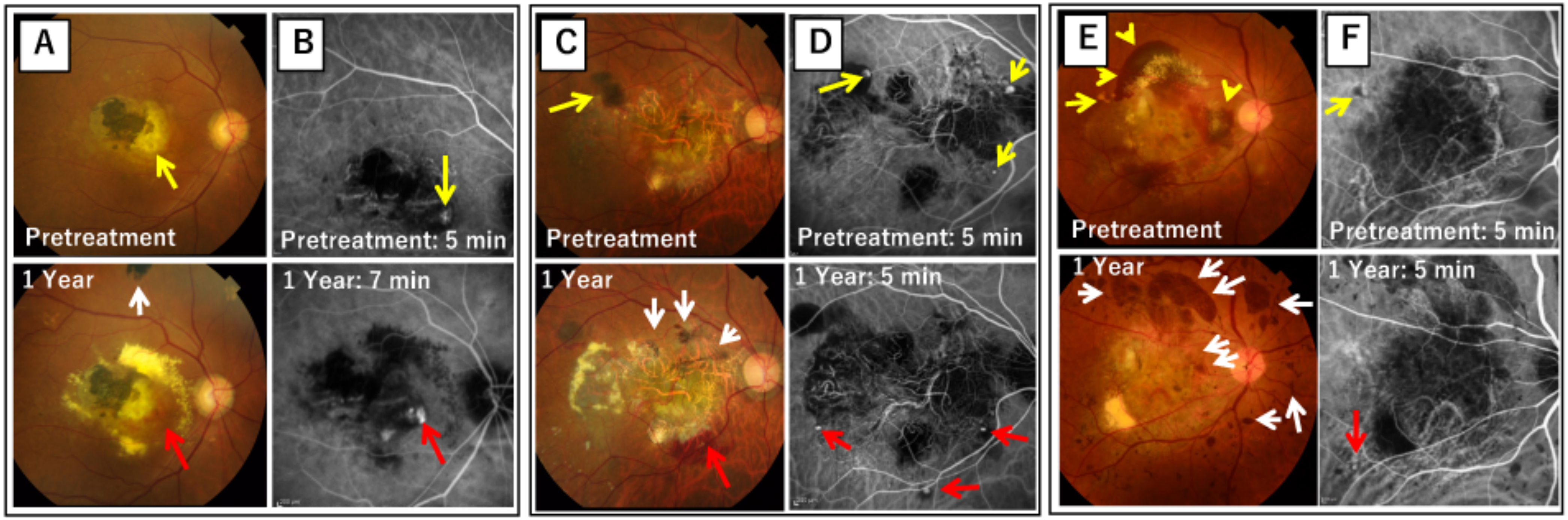

Fig. S4. Color fundus and IA images of before and 1 year after RPE transplantation in Case 3, 4, and 5.

Fundus images of Case 3 (**A**), Case 4 (**C**), and Case 5 (**E**) before and 1 year after iPSC-RPE transplantation. Pigmented clumps or sheets were observed with some on the retinal surface 1 year after transplantation (white arrows) mostly outside the AMD lesion. In IA images (**B, D, F**), yellow arrows indicate the presence of polyp lesions before treatment, and polyp lesions at 1 year after transplantation are marked by red arrows. In Case 5, preoperative polyp lesion (yellow in **F**) caused a hemorrhagic change immediately before surgery (yellow arrowheads, color fundus image), which were stabilized without hemorrhages at 1 year after transplantation.

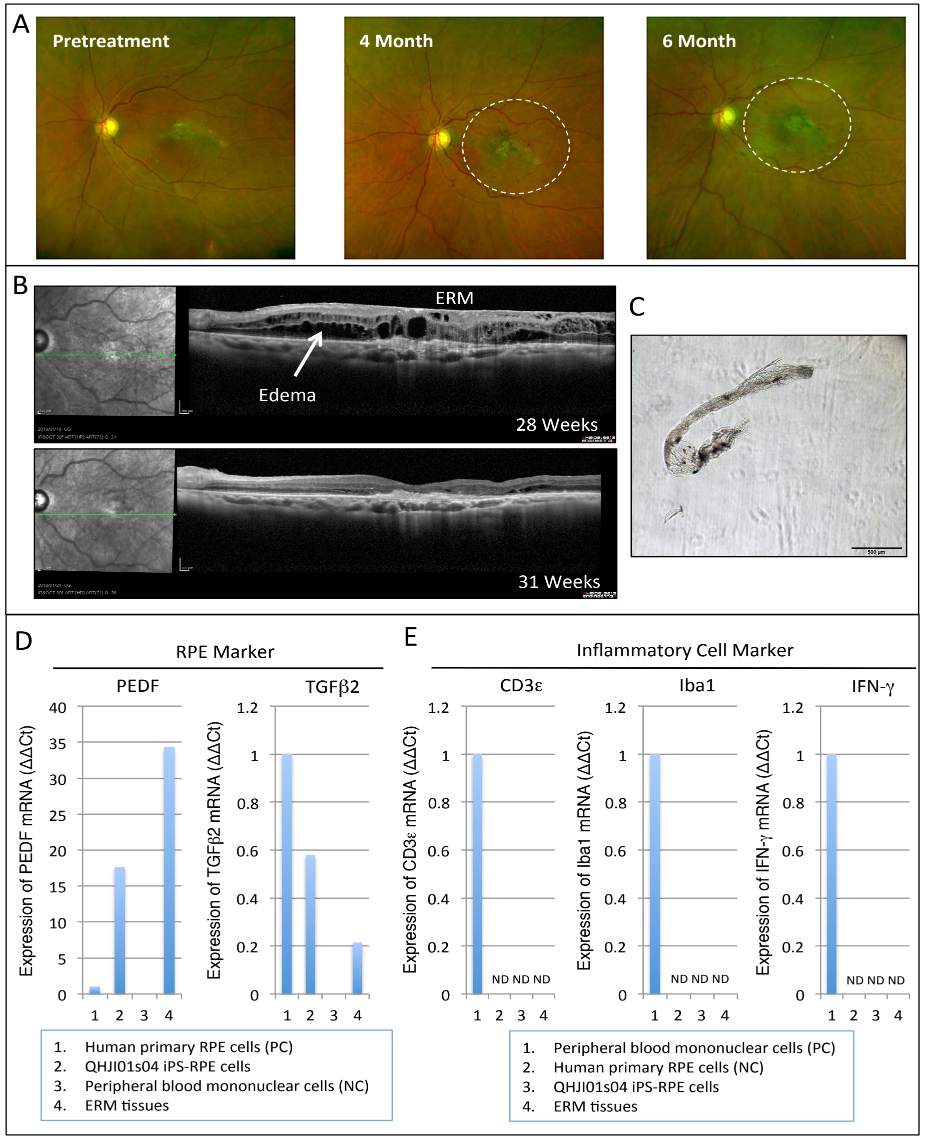

Fig. S5. Evaluation of epiretinal membrane (ERM) from a transplanted AMD patient, Case 2.

Panel **A** shows color fundus photographs for pretreatment, and at 4 or 6 months after surgery of the transplanted AMD patient (Case 2). The circles indicate the ERM on the macular area. Panel **B** shows the OCT images at pretreatment and at 6 months after surgery. We were able to see the diffuse retinal edema together with the ERM on the macular area in Case 2 at 28 weeks. Lower panel in OCT shows removal of the ERM at 31 weeks after transplantation with prompt resolution of the retinal edema. Panel **C** shows color photographs of the removed ERM that contained pigmented tissues. In addition, quantitative RT-PCR determined that the removed ERM tissues contained inflammatory cells. For the assay, we extracted RNA from the ERM tissue. Results showed that the ERM contained RPE markers such as PEDF and TGFβ2, as well as human primary RPE cells (PC: positive control) and cultured iPS-RPE cells, but not peripheral blood cells (NC: negative control: panel **D**). On the other hand, these tissues did not contain inflammatory cells such as CD3e, Iba1, and IFN-γ like human primary RPE cells (NC: panel **E**), which suggests that immune rejection and inflammatory factors may not be relevant with regard to the outcome. Although RT-PCR showed that the removed ERM contained pigmented cells and was positive for RPE markers, the tissues did not contain any inflammatory cells/factors. This suggests that the ERM was of graft cell origin and that the immune rejection may not have been specifically relevant in the outcome.

Fig. S6. Evaluation of grafted iPS-RPE cells by polarization-sensitive OCT (PS-OCT).

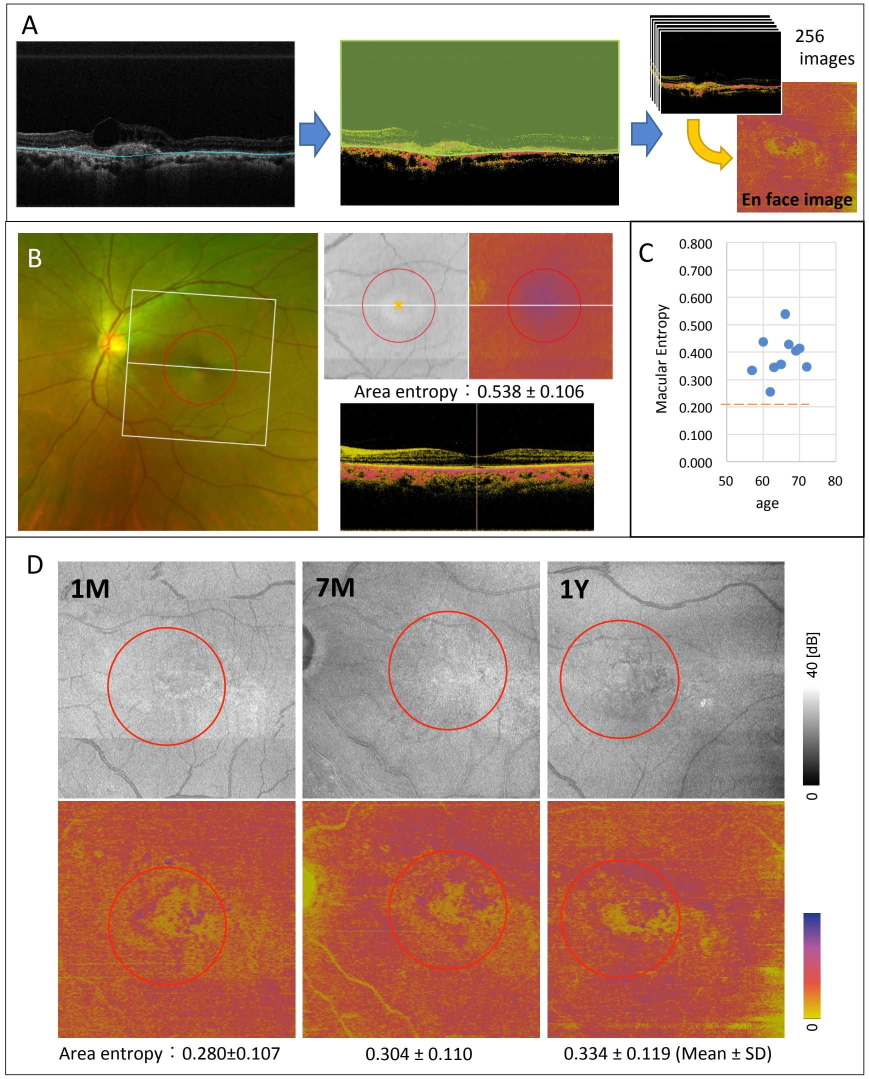

(**A**) By drawing the border line at Bruch's membrane, this made it possible to extract the highest entropy value from the upper retina-RPE area (green shaded area) thereby excluding the choroid area (left 2 panels). As a result, the *en face* image was then created from 256 serial sectional images (right panel). (**B**) The foveal center was determined for the *en face* image and the sectional images. This is indicated by the yellow cross on the *en face* projection image of the OCT intensity and the orange vertical line on the PS-OCT entropy image. The circular region centered at the fovea with a 3-mm diameter was used for the analysis. (**C**) Mean entropy of the circular region was plotted for 10 healthy volunteers (age 65.1±4.4 years) against age. (**D**) Mean entropy of the circular region increased over the measured periods of time after transplantation

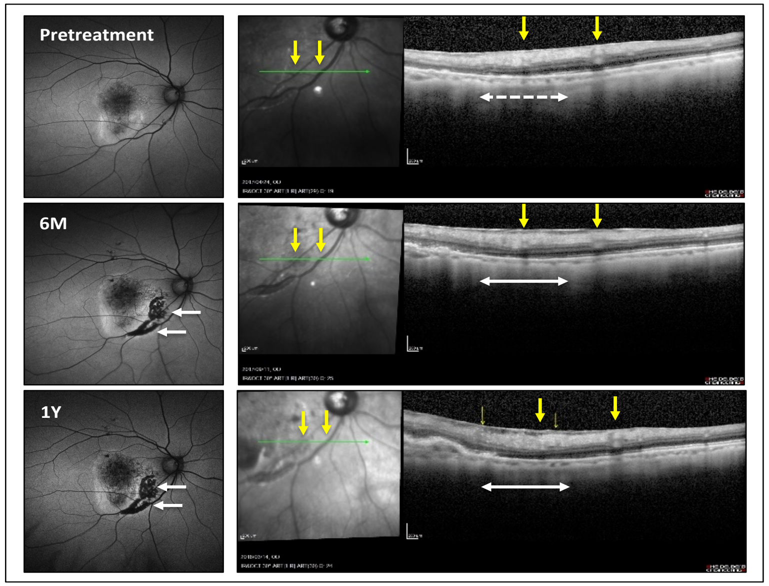

Fig. S7. The presence of transplanted cells did not affect the overlying photoreceptors.

In Case 1, there was a substantial amount of pigmented cells observed on the outside margin of the PCV lesion (see color photo in **Fig. 3**). There was also evidence of these cells in the auto-fluorescence images (white arrows). On the sectional view of the OCT images, the photoreceptor layer on the transplanted RPE cell was not affected and was present and stable at 1 year after transplantation. The vessel shadows correspond to the retinal vessels on each of the fundus images and are indicated by yellow arrows, while the bidirectional white arrows indicate the grafted area. The dotted arrow indicates the same area before transplantation.

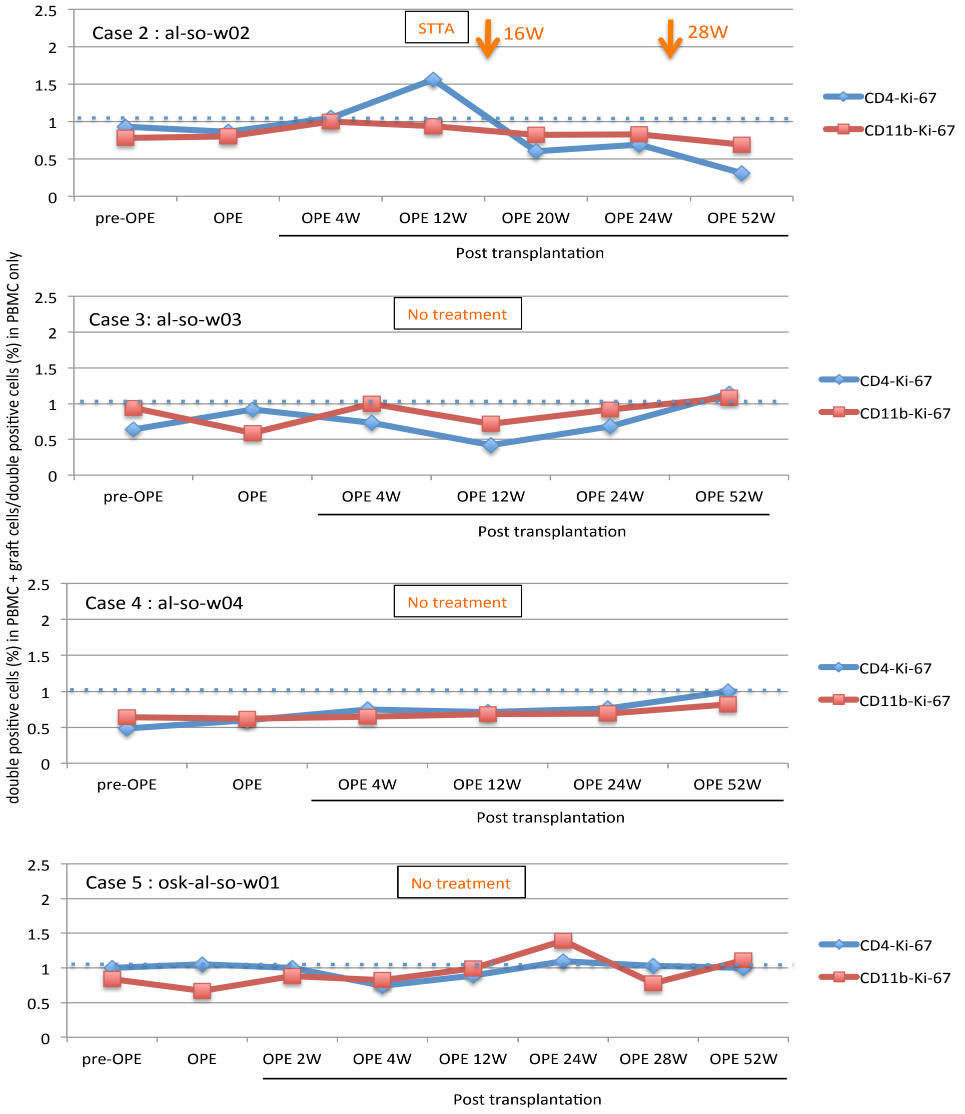

Fig. S8. LGIR FACS results after surgery in the AMD patients, Cases 2, 3, 4 and 5.

Cases 2, 3, 4, and 5 did not exhibit any rejection signs in the eye.

1st graph, Case 2: As $CD4^+/Ki\text{-}67^+$ double-positive cells in PBMC were increased at 12 weeks, we performed sub-Tenon conjunctival injection of triamcinolone (STTA). After treatment, there was a dramatic decrease in the T cells. Subsequently, this patient received STTA therapy twice (at 16 and 28 weeks). 2nd graph, Case 3: There was no increase in the $CD4^+/Ki\text{-}67^+$ or $CD11b^+/Ki\text{-}67^+$ double-positive cells in PBMC during the yearlong follow-up. Therefore, we did not administer any anti-inflammatory medications including STTA for this patient. 3rd graph, Case 4: During the yearlong follow-up, LGIR tests were negative, and thus, this patient also did not undergo any further treatment as well. 4th graph, Case 5: Although $CD11b^+/Ki\text{-}67^+$ double-positive cells in PBMC temporally increased at 24 weeks, we did not perform STTA therapy as this case had triamcinolone-related ocular inflammation after surgery (see **Fig. S10**). During the observation of the eye without treatment, $CD11b^+/Ki\text{-}67^+$ double-positive cells decreased at 28 weeks.

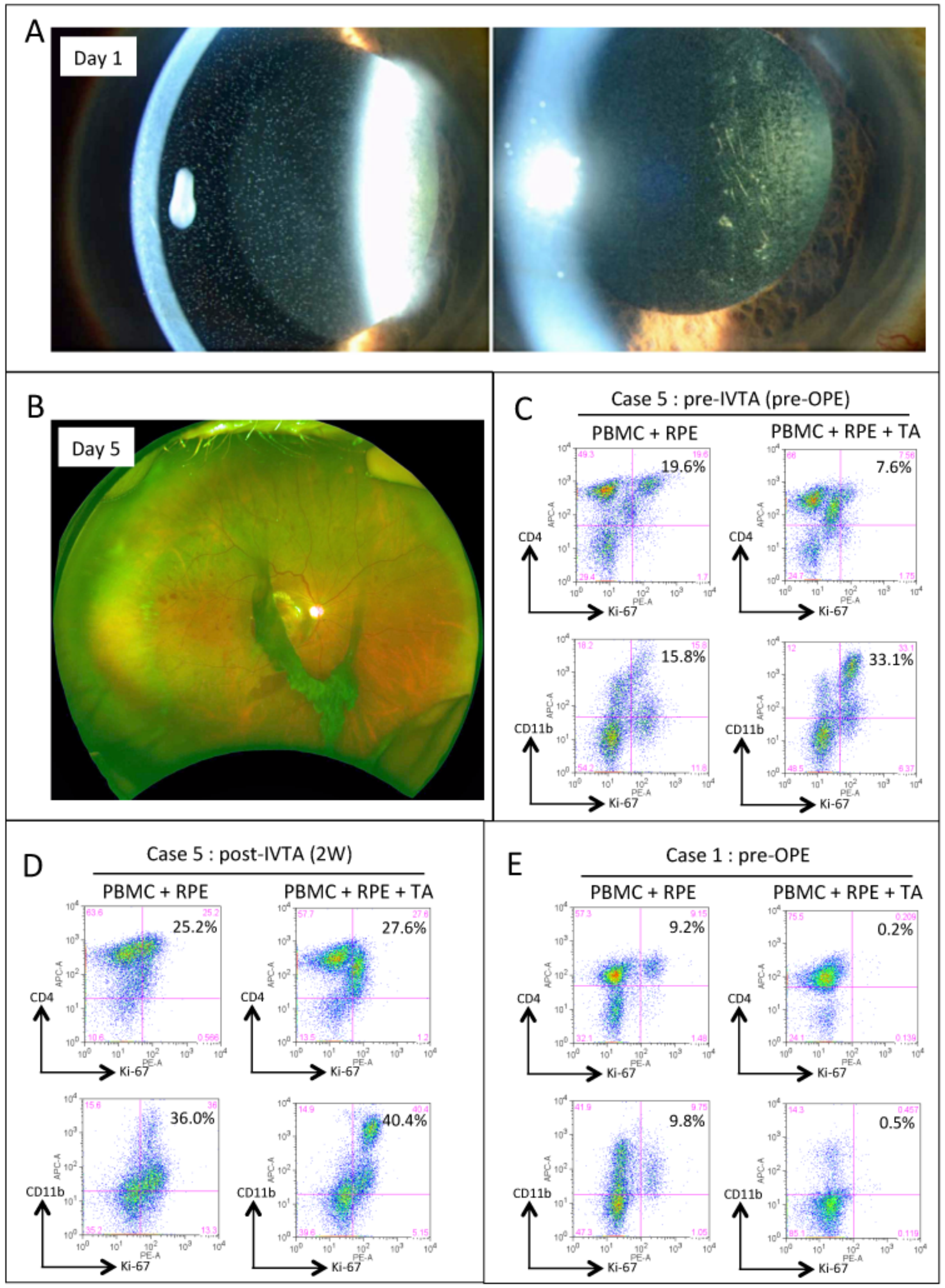

Fig. S9. Sterile endophthalmitis after intravitreal triamcinolone in a transplanted AMD patient.

Panel **A** shows slit lamp photographs at one day after surgery in the transplanted patient (Case 5). Severe anterior chamber cells (left panel) and anterior vitreous cells (right) are seen on the day after surgery. The fundus in the right eye was invisible. Panel **B** shows color fundus photograph at five days. Inflammatory vitreous opacity is observed in the eye. Panel **C** shows the FACS results before surgery in the AMD patient. We performed LGIR test using the patient's blood cells, graft RPE cells, along with triamcinolone (TA) *in vitro*. For the test, $CD4^+$/Ki-67^+ (proliferative helper T cells) and $CD11b^+$/Ki-67^+ (proliferative monocytes) were evaluated using flow cytometry. As compared to PBMC plus RPE cells, the $CD4^+$ T cells in PBMC that were exposed to transplanted iPS-RPE cells in the presence of TA were poorly proliferated. On the other hand, there was a great proliferation of the $CD11b^+$ cells in PBMC. Numbers (%) in the histogram indicate double-positive cells (e.g., $CD4^+$/Ki-67^+). Panel **D** shows the FACS results after surgery in the patient (Case 5). In the test, there was a great proliferation of both $CD4^+$ and $CD11b^+$ cells in PBMC. In addition, there was also an increase in the cytotoxic T cells, B cells, and NK cells (data not shown) after surgery. Panel **E** shows the FACS results for the patient, Case 1. For the LGIR test, there was a great decrease observed in both the $CD4^+$ and $CD11b^+$ cells in PBMC, which suggests that TA might be helping to treat this patient's inflammation. In fact, STTA administration proved to be effective for RPE-related immune rejection for this patient. Therefore, we concluded that the changes in the Case patient were due to TA-related endophthalmitis after IVTA administration.

Table S1. Inclusion and exclusion criteria in our clinical study.

**Inclusion Criteria**
Exudative AMD in at least one eye
Age 50 or over and 85 or under at the time of informed consent
Subfoveal CNV, scar tissue, or RPE tear but without the need to remove CNV
BCVA worse than 0.3* and equal to or better than hand motion (*Equivalent to 20/66 by Snellen chart)
Insufficient effect of anti-VEGF treatments
With or without fluorescein leakage from CNV by fluorescein angiography
Able to understand and willing to sign the informed consent form
Having the HLA haplotype A*24:02, B*52:01, C*12:02, DRB1*15:02, DQB1*06:01, DPB1*09:01
**Exclusion Criteria**
Presence of infectious eye disease
Presence of retinal disease other than AMD
Presence of optic nerve atrophy
Presence of glaucoma with uncontrolled intraocular pressure
Severe liver disorder (AST or ALT higher than 100 IU/L)
Severe renal dysfunction requiring artificial dialysis
Positive for HBV Ag, HCV Ag, HIV antibody, ATL antibody, or serological reaction of syphilis
Allergic to penicillin, streptomycin, or bovine serum
Receiving anticoagulation therapy that cannot be discontinued
History of malignancy except for carcinoma in situ within the last 3 years
Allergic to indocyanine green or fluorescein
Pregnancy (including the possibility) or lactation
Participation in other clinical trial within previous 1 month
Other condition that limits patient compliance, patient safety, or alters study results

HBV Ag - hepatitis B antigen, HCV Ag - hepatitis C antigen, ATL- Adult T-cell lymphoma

Table S2. Number of SNVs and indels and CNVs found in iPSC and RPE cells derived from QHJI01s04.

			**No. of SNVs and indels**			**No. of CNVs***
**Assay**	**Control**	**Sample name**	**on CDS or splicing regions**	**with cosmic 74 IDs**	**listed in Census + Shibata list**	**after filtering**
Whole genome sequencing	Origin	RPE-QHJI01s04_P11_P3	9	2	-	0
	Origin	RPE-QHJI01s04_P12_P3	15	7	-	0
Exome sequencing	Origin	RPE-QHJI01s04_P11_P3	8	2	-	NT
	Origin	RPE-QHJI01s04_P12_P3	7	1	-	NT
Whole genome sequencing	Origin	QHJI01s04_No01_P12	7	1	-	0
	Origin	QHJI01s04_No35_P12	10	4	-	0
	Origin	QHJI01s04_No70_P12	7	2	-	0

*We performed whole genome sequencing and SNP array experiments. -: not found, NT: not tested.

Table S3. Annotation table of SNVs and indels in iPSC and RPE cells derived from QHJI01s04.

	Whole genome sequencing				Exome sequencing				Whole genome sequencing					
	RPE-QHJI01s04_P11_P3		RPE-QHJI01s04_P12_P3		RPE-QHJI01s04_P11_P3		RPE-QHJI01s04_P12_P3		QHJI01s04_No1_P12		QHJI01s04_No35_P12		QHJI01s04_No70_P12	
**Gene name**	Genomon	Genomon2	Genomon	Genomon2	Genomon	Genomon2	Genomon	Genomon2	Genomon	Genomon2	Genomon	Genomon2	Genomon	Genomon2
PTPN5	x: found.	x	x	x	x	x	x	x	x	x	x	x	x	x
NPHS1	x	x	x	x	x	x	x	x	x	x	x	x	x	x
FASTKD1	x	x	x	x	x	x	x	x	x	x	x	x	x	x
EXOC2	x	x	x	x	x	x	x	x	x	x	x	x	x	x
GDPD2	x	x	x	x	x	x	x	x	x	x	x	x	x	x
C7orf72	x	x	x	x	x	x	x	x	x	x	x	x	x	x
RPTN	x		x								x	x		
RP1L1	x		x											
ANKRD12		x												
MUC21			x											
OR2T33			x											
ZNF417			x											
MUC21			x											
ZC3HAV1				x	x	x	x	x						
MUC19				x										
FAM186A				x										
PLG					x							x	x	x
ZNF76									x	x		x		
MUC4												x		

Table S3. *continued*

Gene name	Chr	Start	End	Ref	Alt	Func.refGene	ExonicFunc.refGene	Amino_Change
PTPN5	chr11	18750552	18750552	C	T	Exonic	nonsynonymous SNV	p.E487K,p.E511K,p.E519K,p.E543K
NPHS1	chr19	36317454	36317454	C	T	Exonic	nonsynonymous SNV	p.D1230N
FASTKD1	chr2	170394547	170394547	G	A	Exonic	nonsynonymous SNV	p.R684C
EXOC2	chr6	633058	633058	T	C	Exonic	nonsynonymous SNV	p.I60V
GDPD2	chrX	69649509	69649509	C	T	Exonic	nonsynonymous SNV	p.T289I,p.T368I
C7orf72	chr7	50135885	50135885	C	-	Exonic	frameshift deletion	p.H68fs
RPTN	chr1	152128232	152128232	T	C	Exonic	nonsynonymous SNV	p.N448S
RP1L1	chr8	10465078	10465078	A	G	Exonic	nonsynonymous SNV	p.L2177S
ANKRD12	chr18	9256082	9256082	T	A	Exonic	nonsynonymous SNV	p.D916E,p.D939E
MUC21	chr6	30954806	30954806	T	C	Exonic	nonsynonymous SNV	p.V285A
OR2T33	chr1	248436857	248436857	C	T	Exonic	nonsynonymous SNV	p.S87N
ZNF417	chr19	58421080	58421080	G	T	Exonic	nonsynonymous SNV	p.A189E,p.A188E
MUC21	chr6	30954909	30954909	C	G	Exonic	nonsynonymous SNV	p.D319E
ZC3HAV1	chr7	138732476	138732476	G	A	Exonic	nonsynonymous SNV	p.T858M
MUC19	chr12	40879656	40879656	A	G	Exonic	unknown	UNKNOWN
FAM186A	chr12	50746740	50746740	T	G	Exonic	nonsynonymous SNV	p.N1292T
PLG	chr6	161139775	161139775	G	A	Exonic	stopgain SNV	p.W334X
ZNF76	chr6	35258145	35258145	G	T	Exonic	nonsynonymous SNV	p.A179S
MUC4	chr3	195512767	195512767	T	G	Exonic	nonsynonymous SNV	p.N1895T

Table S3. *continued*

Gene name	genomicSuperDups	Cancer_gene_Census	Shibata list	cosmic74_position (occurrence)	hgmd2015.3	snp131	snp138	esp6500si_all	1000g2014oct_all	HGVDratio
PTPN5	-	-	-	-	-	-	-	-	-	-
NPHS1	-	-	-	1	-	-	-	-	-	-
FASTKD1	-	-	-	-	-	-	rs369748449	0.000077	-	-
EXOC2	-	-	-	-	-	-	-	-	-	-
GDPD2	-	-	-	-	-	-	-	-	-	-
C7orf72	-	-	-	-	-	-	-	-	-	-
RPTN	-	-	-	1	-	-	rs200878903	-	-	-
RP1L1	-	-	-	-	-	-	-	-	-	-
ANKRD12	-	-	-	-	-	-	-	-	-	-
MUC21	-	-	-	1	-	rs9262370	rs9262370	-	-	-
OR2T33	Score=0.931148	-	-	9	-	rs75662887	rs75662887	-	-	-
ZNF417	Score=0.936519	-	-	7	-	rs3810130	rs3810130	-	0.0998403	-
MUC21	-	-	-	5	-	rs9262380	rs9262380	-	-	-
ZC3HAV1	-	-	-	-	-	-	-	-	-	-
MUC19	-	-	-	-	-	-	-	-	-	-
FAM186A	-	-	-	5	-	-	-	-	-	-
PLG	-	-	-	1	-	-	-	-	-	-
ZNF76	-	-	-	-	-	-	-	-	-	-
MUC4	Score=0.920296	-	-	12	-	rs2641778	rs2641778	-	-	-

-: not found.

Table S4. Alternative allele frequencies of SNVs and indels of Origin, iPSC and RPE cells derived from QHJI01s04.

	Whole genome sequencing						Exome sequencing					
	Origin		RPE-QHJI01s04_P11_P3		RPE-QHJI01s04_P12_P3		Origin		RPE-QHJI01s04_P11_P3		RPE-QHJI01s04_P12_P3	
Gene	Total	Alt. allele freq.	Total	Alt. allele freq.	Total	Alt. allele freq.	Total	Alt. allele freq.	Total	Alt. allele freq.	Total	Alt. allele freq.
PTPN5	79	0.0%	63	36.5%	63	44.4%	81	3.7%	53	43.4%	33	60.6%
NPHS1	65	0.0%	75	44.0%	67	40.3%	90	0.0%	50	40.0%	57	54.4%
FASTKD1	100	1.0%	100	48.0%	85	50.6%	139	0.0%	259	38.2%	216	42.6%
EXOC2	76	0.0%	127	55.9%	93	48.4%	136	0.0%	105	48.6%	81	55.6%
GDPD2	38	0.0%	37	100.0%	28	100.0%	49	0.0%	56	100.0%	38	100.0%
C7orf72	94	0.0%	94	38.3%	105	46.7%	115	0.0%	189	47.6%	125	36.8%
RPTN	75	1.3%	95	20.0%	79	22.8%	477	2.3%	309	1.0%	269	1.9%
RP1L1	100	2.0%	102	15.7%	75	16.0%	75	2.7%	69	1.4%	56	0.0%
ANKRD12	66	0.0%	115	7.8%	110	0.0%	23	0.0%	97	2.1%	92	0.0%
MUC21	83	2.4%	84	26.2%	62	41.9%	92	6.5%	44	0.0%	36	0.0%
OR2T33	77	6.5%	91	36.3%	73	45.2%	303	17.2%	243	10.3%	195	17.9%
ZNF417	31	0.0%	58	17.2%	60	36.7%	64	0.0%	54	0.0%	43	0.0%
MUC21	72	1.4%	62	22.6%	85	29.4%	75	4.0%	46	0.0%	37	2.7%
ZC3HAV1	66	0.0%	87	8.0%	82	11.0%	117	0.0%	197	9.6%	145	11.0%
MUC19	70	1.4%	93	10.8%	91	15.4%	4	0.0%	0	0.0%	0	0.0%
FAM186A	88	5.7%	68	25.0%	77	32.5%	187	2.1%	124	0.8%	114	3.5%
PLG	78	0.0%	94	5.3%	87	4.6%	73	0.0%	136	12.5%	110	12.7%
ZNF76	67	0.0%	75	2.7%	70	5.7%	79	0.0%	84	3.6%	55	9.1%
MUC4	149	4.0%	147	17.7%	176	22.7%	543	1.8%	285	1.1%	253	1.2%

Table S4. *continued*

	Whole genome sequencing								Amplicon Sequencing					
	Origin		QHJI01_33_No1_P12		QHJI01_33_No35_P12		QHJI01_33_No70_P12		Origin		RPE-QHJI01s04_P11_P3		RPE-QHJI01s04_P12_P3	
Gene	Total	Alt. allele freq.	Total	Alt. allele freq.	Total	Alt. allele freq.	Total	Alt. allele freq.	Total	Alt. allele freq.	Total	Alt. allele freq.	Total	Alt. allele freq.
PTPN5	46	2.2%	40	60.0%	59	67.8%	53	60.4%	91891	1.7%	96516	52.1%	95007	49.5%
NPHS1	55	0.0%	64	45.3%	60	50.0%	64	40.6%	99839	0.5%	34727	48.5%	43004	49.8%
FASTKD1	82	0.0%	100	49.0%	71	33.8%	81	51.9%	1351	0.4%	526	58.2%	559	55.1%
EXOC2	75	0.0%	83	61.4%	63	44.4%	100	46.0%	63838	0.1%	9206	49.7%	12779	49.1%
GDPD2	20	0.0%	27	100.0%	32	100.0%	46	97.8%	24375	3.8%	6633	98.9%	7560	99.1%
C7orf72	87	0.0%	86	46.5%	55	56.4%	72	52.8%	589	1.4%	37	70.3%	41	51.2%
RPTN	73	5.5%	80	18.8%	60	25.0%	85	9.4%	14654	37.4%	67607	49.8%	64583	49.8%
RP1L1	62	6.5%	78	12.8%	94	8.5%	96	11.5%	not tested	not tested	not tested	not tested	not tested	not tested
ANKRD12	124	0.0%	71	2.8%	65	3.1%	63	1.6%	9712	0.0%	2588	0.2%	2993	0.2%
MUC21	48	6.3%	88	10.2%	89	21.3%	76	22.4%	11428	22.0%	36302	17.6%	30867	17.6%
OR2T33	71	23.9%	63	27.0%	58	43.1%	65	29.2%	38926	39.8%	98396	36.2%	69008	36.4%
ZNF417	39	10.3%	50	32.0%	58	27.6%	51	29.4%	not tested	not tested	not tested	not tested	not tested	not tested
MUC21	36	0.0%	84	20.2%	70	35.7%	93	11.8%	18710	19.1%	53222	16.1%	45292	16.5%
ZC3HAV1	79	0.0%	73	6.8%	68	8.8%	91	5.5%	115807	0.1%	24354	9.0%	31511	8.3%
MUC19	84	3.6%	80	8.8%	75	12.0%	81	8.6%	not tested	not tested	not tested	not tested	not tested	not tested
FAM186A	29	6.9%	80	18.8%	98	13.3%	104	13.5%	1577	50.9%	6192	49.3%	4713	55.6%
PLG	100	0.0%	79	7.6%	64	9.4%	91	11.0%	22544	0.0%	5948	4.8%	6915	8.0%
ZNF76	56	0.0%	56	21.4%	70	11.4%	57	12.3%	119315	0.1%	33410	5.2%	40150	6.9%
MUC4	96	4.2%	137	10.9%	158	15.8%	142	8.5%	not tested	not tested	not tested	not tested	not tested	not tested

Table S5. Sequencing statistics of whole genome sequencing and exome sequencing.

	Whole genome sequencing			Exome sequencing			Whole genome sequencing			
**Sample Name**	**Origin**	**RPE-QHJI01s04_P11_P3**	**RPE-QHJI01s04_P12_P3**	**Origin**	**RPE-QHJI01s04_P11_P3**	**RPE-QHJI01s04_P12_P3**	**Origin**	**QHJI01s04_No1_P12**	**QHJI01_s04_No35_P12**	**QHJI01_s04_No70_P12**
Library prep kit	KAPA Hyper Prep (PCR Free)			Nimblegen SeqCap EZ Library SR v3.0			Nextera DNA			
Sequence length	126bp, PE	126bp, PE	126bp, PE	126bp, PE	126bp, PE	126bp, PE	126bp, PE	126bp, PE	126bp, PE	126bp, PE
Sequencing coverage	70.52	82.75	79.62	98.46	106.92	87.08	63.66	64.98	60.94	67.06
No. of sequencing reads	2239.5M	2326.8M	2241.4M	89.4M	104M	86M	1907M	1865.9M	1744M	1933M
No. of mapped reads	2089.7M	2207M	2125.3M	86.5M	100M	83.9M	1714M	1720.3M	1607.8M	1776.7M
Mapped rate (%)	93.3	94.9	94.8	96.8	96.2	97.6	89.9	92.2	92.2	91.91

M: million

Table S6. Primers used in Amplicon sequencing to validate SNVs found in iPSC and RPE cells.

Gene name	Forward Primer	Reverse Primer
PTPN5	CCCAGAGAACCTTGTAGGAGAAG	GATTGCTACTCCATGGCTTTTGG
NPHS1	ATGAGAGAGACCAGTGGAGTGTA	GGCCTGAAGACACATATCAGGAT
FASTKD1	GGAAGGCTCGTGTCATGTTTAAG	ATCTCCATCTCGAAGTGCAAGAG
EXOC2	AAGAGACTGTTGAGGTTCCTCTG	TGTGGACATAATTGCCTCCTGAC
GDPD2	GTCAGACGGTACCAGCATTAGAA	GCAGCCTCAAAATCATCCTCATC
C7orf72	GAGAACAGACATAACTATGGCAGG	CCAGGACTCATAAACCCTGAACA
RPTN	TCTGACCATAGTGGGAACTTTGG	GTCAACCAAACAGACAAGGTCAG
ANKRD12	CCTGAAAAAGAGAGGCATCTAGC	CCTTTTTATCTAGCTCCCTGTC
MUC21	GAGTCCAGAACGACCTCCAATG	TTGTGCTGGAGTCAGAGTTGG
OR2T33	TATGGGAAGCTCAGGGTAACAAC	TACTTCCTCCTGAGCCAACTTTC
MUC21	GAGTCCAGAACGACCTCCAATG	TTGTGCTGGAGTCAGAGTTGG
ZC3HAV1	GAGGGATTCGATCTGGTATCCAC	CGTCGTTATGTTTGTAGCCCAAG
FAM186A	GTGAGAGTGATCTCCTGAGTCTG	GATCACTCTAACCCTTCAGCAGG
PLG	GTAACGGTTGTTCTCAAAGCGTG	TAAGCTCCCATACCCTTGCTTAC
ZNF76	AGATTCCCCGTAATGGAAAAGGG	GTACCCTCATTTCTTCCCTCCAG

RPE cells were derived from a QHJI01s04 donor.

Table S7. Results of residual vector test in iPS cells.

Sample name	Target	Ct value			
		**P11**	**P12**	**P13**	**P14**
QHJI01s04	KLF4	N/D	N/D	N/D	N/D
	MYCL1	N/D	N/D	N/D	N/D
	Trp53	N/D	N/D	N/D	N/D
	LIN28A	43.986	40.913	N/D	N/D
	SOX2	N/D	N/D	N/D	N/D
	OCT3/4	N/D	N/D	N/D	N/D
	EBNA1	N/D	N/D	N/D	N/D
	WPRE	N/D	N/D	N/D	N/D

P; passage, N/D; not detected

Table S8. Results of quality control tests in iPS-RPE cells.

No.	Clone	Morphology	Cell Viability (%, n=3)	RPE Marker (RT-PCR)	RPE Purity-1 Best1-Pax6 (%) MiTF-Pax6 (%)	RPE Purity-2 Adhesion test (%)	iPS cells Marker *LIN28* (qRT-PCR)	Infectious Pathogens (qPCR)	Mycoplasma (Sterility Test) (PCR)	Sterility Test	Endotoxin (EU/mL)	HLA Haplotype
1	QHJI01s01-RPE-01	Passed	91.0	Positive	99.6<br>99.6	0.140	Negative	Negative	Negative<br>Negative	Negative	0.52	Same as iPS cells
2	QHJI01s01-RPE-02	Passed	87.9	Positive	99.1<br>99.6	0.144	Negative	Negative	Negative<br>Negative	Negative	0.40	Same as iPS cells
3	QHJI01s04-RPE-01	Passed	93.4	Positive	99.6<br>99.6	0.187	Negative	Negative	Negative<br>Negative	Negative	0.39	Same as iPS cells
4	QHJI01s04-RPE-02	Passed	90.1	Positive	100<br>98.7	0.116	Negative	Negative	Negative<br>Negative	Negative	0.35	Same as iPS cells

We tested 4 iPS-RPE cell lines for the quality standard and assessment. All cells passed the tests, and thus, we finally picked QHJI01s04-RPE-01 cells for the transplantation. In addition, *in vivo* tumorigenicity tests were all negative in these 4 lines (see **Fig. S3**). Specific details for each of the tests are described in our previous report.[8]

Table S9. Results of quality control tests in iPS-RPE cells on transplantation day.

Case	Transplanted Cells	Transplantation Day<br>Cell Culture Day	Cell Viability (%)<br>SV: >70%	Cell Density<br>SV: 2 x 10^6 -1 x 10^7	Infectious Pathogens<br>SV: Not detected	Mycoplasma<br>SV: Not detected	Sterility Test<br>SV: Not detected	Endotoxin (EU/mL)<br>SV: <3 EU/mL
1	QHJI01s01-RPE-01-13, 14	3/28/2017<br>3/14/2017	91.0	5.2 x 10^6	Negative	Negative	Negative	0.41
2	QHJI01s01-RPE-01-15, 16, 17	6/27/2017<br>6/13/2017	91.1	4.2 x 10^6	Negative	Negative	Negative	0.32
3	QHJI01s01-RPE-01-18, 19, 20	7/25/2017<br>7/11/2017	98.2	4.82 x 10^6	Negative	Negative	Negative	0.40
4	QHJI01s01-RPE-01-21, 22, 23	9/12/2017<br>8/29/2017	84.4	4.95 x 10^6	Negative	Negative	Negative	0.297
5	QHJI01s01-RPE-01-27, 28, 29	9/21/2017<br>9/7/2017	88.8	4.28 x 10^6	Negative	Negative	Negative	0.285

On surgery day, we tested iPS cells-derived RPE cells for the quality standard and assessment. All cells passed these tests. Thus, we used the RPE cells for the transplantation in each patient.
SV - Standard value.

Table S10. Summary of immunological tests in transplanted AMD patients - LGIR tests.

No.	Case	Pre-OPE	OPE	2W	4W	8W	12W	20W	24W	28W	36W	52W	Tested PBMC
1	al-so-w01	−	−	nt	+	+	±	nt	±	nt	−	−	n=8
2	al-so-w02	−	−	nt	−	nt	±	−	−	nt	nt	−	n=7
3	al-so-w03	−	−	nt	−	nt	−	nt	−	nt	nt	−	n=6
4	al-so-w04	−	−	nt	−	nt	−	nt	−	nt	nt	−	n=6
5	osk-al-so-w01	−	−	−	−	nt	−	nt	±	−	nt	−	n=8

Plus (+) indicates LGIR tests were positive, plus-minus (±) indicates LGIR tests were suspected positive, and minus (-) indicates LGIR tests were negative.

Pre-OPE (pre-operation) and OPE (operation day) indicate data before transplantation. nt - not tested, PBMC - peripheral blood mononuclear cells.

Table S11. Summary of immunological tests in transplanted AMD patients – RSA tests.

No.	Case	Pre-OPE	OPE	2W	4W	8W	12W	20W	24W	28W	36W	52W	Tested serum
1	al-so-w01	−	−	nt	−	−	−	nt	−	nt	−	−	n=8
2	al-so-w02	−	−	nt	−	nt	−	−	−	nt	nt	−	n=7
3	al-so-w03	−	−	nt	−	nt	−	nt	−	nt	nt	−	n=6
4	al-so-w04	−	−	nt	−	nt	−	nt	−	nt	nt	−	n=6
5	osk-al-so-w01	−	−	−	−	nt	−	nt	−	−	nt	−	n=8

Plus (+) indicates RSA tests were positive, plus-minus (±) indicates RSA tests were suspected positive, and minus (-) RSA tests were negative.

All tested samples from AMD patients were negative in this study. Pre-OPE (pre-operation) and OPE (operation day) indicate data before transplantation.

nt - not tested.

Table S12. Summary of secondary outcome in this study.

**Case 1: al-so-w01**	**Pre-OPE**	**52W**
Foveal retinal thickness (OCT: μm)	133	89
Subretinal fluid (OCT)	Yes	Yes (Decrease)
Retinal edema (OCT)	Yes	No
Retinal sensitivity: Multi-focal ERG	10.9	6.9
Retinal sensitivity: Microperimetry (MP3: dB)	10.97	8.3
BCVA	0.08 (20/250)	0.06 (20/320)
Dye leakage from CNV (FA: μm)	4.114	3.74
Number of anti-VEGF treatment (/year)	4	3
QOL (VFQ-25 scores)	28.5	42.1

**Case 2: al-so-w02**	**Pre-OPE**	**52W**
Foveal retinal thickness (OCT: μm)	123	428
Subretinal fluid (OCT)	No	No
Retinal edema (OCT)	No	Yes (Increase)
Retinal sensitivity: Multi-focal ERG	7.44	6.04
Retinal sensitivity: Microperimetry (MP3: dB)	16.69	13.23
BCVA	0.1 (10/100)	0.15 (20/125)
Dye leakage from CNV (FA: μm)	2.332	1
Number of anti-VEGF treatment (/year)	2	3
QOL (VFQ-25 scores)	82.6	93.9

**Case 3: al-so-w03**	**Pre-OPE**	**52W**
Foveal retinal thickness (OCT: μm)	688	544
Subretinal fluid (OCT)	No	Yes (Decrease)

Retinal edema (OCT)	Yes	Yes (Decrease)
Retinal sensitivity: Multi-focal ERG	7.52	5.85
Retinal sensitivity: Microperimetry (MP3: dB)	8.86	5.43
BCVA	0.1 (10/100)	0.15 (20/125)
Dye leakage from CNV (FA: µm)	2.451	2.21
Number of anti-VEGF treatment (/year)	3	2
QOL (VFQ-25 scores)	22.3	27.2

**Case 4: al-so-w04**	**Pre-OPE**	**52W**
Foveal retinal thickness (OCT: µm)	34	21
Subretinal fluid (OCT)	Yes	No
Retinal edema (OCT)	No	Yes (Increase)
Retinal sensitivity: Multi-focal ERG	nt	nt
Retinal sensitivity: Microperimetry (MP3: dB)	2.4	1.3
BCVA	0.09 (10/100)	0.1 (10/100)
Dye leakage from CNV (FA: µm)	8.926	6.57
Number of anti-VEGF treatment (/year)	1	1
QOL (VFQ-25 scores)	92.7	90.1

**Case 5: OSK-al-so-w-01**	**Pre-OPE**	**52W**
Foveal retinal thickness (OCT: µm)	872	620
Subretinal fluid (OCT)	Yes	NO
Retinal edema (OCT)	Yes	Yes (Decrease)
Retinal sensitivity: Multi-focal ERG	8.97	7.94
Retinal sensitivity: Microperimetry (MP3: dB)	4.4	2.4
BCVA	0.2 (20/100)	0.15 (20/125)

Dye leakage from CNV (FA: μm)	7.24	6.344
Number of anti-VEGF treatment (/year)	3	0
QOL (VFQ-25 scores)	95.6	95.2

Data before transplantation were collected at 0 weeks (2nd screening day: Pre-OPE). Data after the transplantation were collected at 52 weeks (52W) after surgery.

Microperimetry data is shown as the retinal sensitivity (mean: dB) of AMD lesions. Multi-focal ERG data is a mean amplitude of AMD lesions.

Dye leakage from CNV measures the greatest linear diameter (GLD) of CNV by FA examination.

BCVA (best-corrected visual acuity) indicates that 0.1 is equivalent to 10/100 on a Snellen chart.

Table S13. Concomitant drugs in all cases.

Subject ID Code	Drug	Administration Route	Dose	Administration Start Date	Administration End Date	Reason for Administration
al-so-w01	Gatifloxacin eyedrops	Instillation (RE only)	4 administrations	March 27, 2017	Continued through 52W	Treatment related to this study
al-so-w01	Betamethasone sodium phosphate eyedrops	Instillation (RE only)	8 administrations	March 28, 2017	April 10, 2017	Treatment of AE
al-so-w01	Cefcapene pivoxil hydrochloride tablets	P.O.	3 tablets	March 29, 2017	March 31, 2017	Treatment related to this study
al-so-w01	Ofloxacin ophthalmic ointment	Instillation (RE only)	1 administration	March 29, 2017	April 30, 2017	Treatment of AE
al-so-w01	Alogliptin benzoate tablets	P.O.	25 mg	April 01, 2017	Continued through 52W	Complication
al-so-w01	Purified sodium hyaluronate	Instillation (RE only)	6 administrations	April 05, 2017	Continued through 52W	Treatment of AE
al-so-w01	d-chlorpheniramine maleate	I.V.	5 mg	June 26, 2017	June 26, 2017	Prophylactic administration (prevent contrast media allergy)
al-so-w01	Methylprednisolone sodium succinate	I.V.	40 mg	June 26, 2017	June 26, 2017	Prophylactic administration (prevent contrast media allergy)
al-so-w01	Methylprednisolone sodium succinate	I.V.	40 mg	September 11, 2017	September 11, 2017	Prophylactic administration (prevent contrast media allergy)

AE - adverse event, RE – right eye.

Concomitant drugs *(continued)*

Subject ID Code	Drug	Administration Route	Dose	Administration Start Date	Administration End Date	Reason for Administration
al-so-w01	d-chlorpheniramine maleate salt	I.V.	5 mg	September 11, 2017	September 11, 2017	Prophylactic administration (prevent contrast media allergy)
al-so-w01	Prednisolone	P.O.	10 mg	April 24, 2017	April 25, 2017	Treatment of AE
al-so-w01	Dexamethasone sodium phosphate	I.V.	3.3 mg	April 24, 2017	April 24, 2017	Treatment of AE
al-so-w01	d-chlorpheniramine maleate	I.V.	5 mg	April 24, 2017	April 24, 2017	Treatment of AE
al-so-w01	d-chlorpheniramine maleate	I.V.	5 mg	September 11, 2017	September 11, 2017	Treatment of AE
al-so-w01	Fexofenadine hydrochloride	P.O.	120 mg	April 24, 2017	April 25, 2017	Treatment of AE
al-so-w01	Fexofenadine hydrochloride	P.O.	120 mg	June 26, 2017	June 27, 2017	Treatment of AE
al-so-w01	Prednisolone	P.O.	10 mg	June 26, 2017	June 27, 2017	Treatment of AE
al-so-w01	Dexamethasone sodium phosphate	I.V.	3.3 mg	September 11, 2017	September 11, 2017	Treatment of AE
al-so-w01	Fexofenadine hydrochloride	P.O.	120 mg	September 11, 2017	September 12, 2017	Treatment of AE
al-so-w01	Prednisolone	P.O.	10 mg	September 11, 2017	September 12, 2017	Treatment of AE
al-so-w01	Tranexamic acid capsule 250 mg	P.O.	750 mg	November 04, 2017	November 08, 2017	Treatment of AE

AE - adverse event.

Concomitant drugs *(continued)*

Subject ID Code	Drug	Administration Route	Dose	Administration Start Date	Administration End Date	Reason for Administration
al-so-w01	Fexofenadine hydrochloride 60 mg	P.O.	120 mg	November 04, 2017	November 08, 2017	Treatment of AE
al-so-w01	Metformin hydrochloride	P.O.	500 mg	January 06, 2018	Continued through 52W after transplantation	Complication
al-so-w01	Methylprednisolone sodium succinate	I.V.	125 mg	March 14, 2018	March 14, 2018	Prophylactic administration (allergy)
al-so-w01	d-chlorpheniramine maleate	I.V.	5 mg	March 14, 2018	March 14, 2018	Treatment of AE
al-so-w01	Dexamethasone sodium phosphate	I.V.	3.3 mg	March 14, 2018	March 14, 2018	Treatment of AE
al-so-w01	Dexamethasone sodium phosphate	I.V.	3.3 mg	March 14, 2018	March 14, 2018	Prophylactic administration (allergy prevention)
al-so-w01	d-chlorpheniramine maleate	I.V.	5 mg	March 14, 2018	March 14, 2018	Prophylactic administration (allergy)
al-so-w01	Betamethasone sodium phosphate eyedrops	Instillation (RE only)	4 administrations	April 11, 2017	Continued through 52W	Treatment related to this study
al-so-w02	Cefcapene pivoxil hydrochloride hydrate	P.O.	200 mg	June 28, 2017	June 30, 2017	Treatment related to this study
al-so-w02	KREMEZIN Fine Granules	P.O.	6 g	Administration began before ENR	Continued through 52W	Complication

ENR – enrollment, AE - adverse event, RE – right eye.

Concomitant drugs *(continued)*

Subject ID Code	Drug	Administration Route	Dose	Administration Start Date	Administration End Date	Reason for Administration
al-so-w02	Cilostazol	P.O.	200 mg	Administration began before ENR	Continued through 52W	Prophylactic administration (prevention of cerebral infarction)
al-so-w02	Doxazosin mesylate	P.O.	3 mg	Administration began before ENR	December 15, 2017	Complication
al-so-w02	Tamsulosin hydrochloride	P.O.	0.2 mg	Administration began before ENR	December 15, 2017	Complication
al-so-w02	Candesartan cilexetil	P.O.	8 mg	Administration began before ENR	Continued through 52W	Complication
al-so-w02	Febuxostat	P.O.	10 mg	Administration began before ENR	Continued through 52W	Complication
al-so-w02	Amlodipine besylate	P.O.	5 mg	Administration began before ENR	Continued through 52W	Complication
al-so-w02	Esomeprazole magnesium hydrate	P.O.	20 mg	Administration began before ENR	Continued through 52W	Other (gastric mucosa protection)
al-so-w02	Epinastine hydrochloride	P.O.	20 mg	Administration began before ENR	Continued through 52W	Complication
al-so-w02	Gatifloxacin hydrate	Instillation (LE only)	4 administrations	June 26, 2017	Continued through 52W	Treatment related to this study
al-so-w02	Betamethasone sodium phosphate	Instillation (LE only)	4 administrations	June 26, 2017	March 28, 2018	Treatment related to this study

ENR – enrollment, LE – left eye.

Concomitant drugs *(continued)*

Subject ID Code	Drug	Administration Route	Dose	Administration Start Date	Administration End Date	Reason for Administration
al-so-w02	Latanoprost	Instillation (LE only)	1 administration	August 14, 2017	August 28, 2017	Treatment of AE
al-so-w02	Latanoprost	Instillation LE only)	1 administration	September 04, 2017	September 20, 2017	Treatment of AE
al-so-w02	Brinzolamide	Instillation (LE only)	2 administrations	August 29, 2017	March 28, 2018	Treatment of AE
al-so-w02	Rinderon-V ointment 0.12%	Topical	Sufficient quantity	December 21, 2017	Continued through 52W	Complication
al-so-w02	Keratinamin kowa cream 20%	Topical	Sufficient quantity	December 21, 2017	Continued through 52W	Complication
al-so-w02	Patell Tape 40	Topical	Sufficient quantity	December 21, 2017	Continued through 52W	Complication
al-so-w02	Sulprotin ointment 1%	Topical	Sufficient quantity	December 21, 2017	Continued through 52W	Complication
al-so-w02	Dermosol G lotion	Topical	Sufficient quantity	January 26, 2018	Continued through 52W	Complication
al-so-w02	Kindalone ointment 0.05%	Topical	Sufficient quantity	February 22, 2018	Continued through 52W	Complication
al-so-w02	Latanoprost	Instillation (LE only)	1 administration	November 22, 2017	Continued through 52W	Treatment of AE
al-so-w02	Brinzolamide	Instillation (LE only)	2 administrations	April 04, 2018	Continued through 52W	Treatment of AE

AE - adverse event, LE – left eye.

Concomitant drugs *(continued)*

Subject ID Code	Drug	Administration Route	Dose	Administration Start Date	Administration End Date	Reason for Administration
al-so-w02	COSOPT ophthalmic solution	Instillation (LE only)	2 administrations	March 28, 2018	April 03, 2018	Treatment of AE
al-so-w02	Fluorometholone	Instillation LE only)	4 administrations	March 29, 2018	Continued through 52W	Treatment related to this study
al-so-w02	Diamox tablets	P.O.	500 mg	March 28, 2018	March 29, 2018	Treatment of AE
al-so-w02	Gluconsan K tablets	P.O.	10 mEq	March 28, 2018	March 29, 2018	Treatment of AE
al-so-w03	Aliskiren fumarate tablets	P.O.	150 mg	Administration began before ENR	Continued through 52W	Complication
al-so-w03	Naftopidil OD tablets	P.O.	25 mg	Administration began before ENR	Continued through 52W	Complication
al-so-w03	Allopurinol	P.O.	100 mg	Administration began before ENR	October 25, 2017	Complication
al-so-w03	Pitavastatin calcium tablets	P.O.	1 mg	Administration began before ENR	October 25, 2017	Complication
al-so-w03	Zolpidem tartrate tablets	P.O.	10 mg	Administration began before ENR	Continued through 52W	Complication
al-so-w03	Etodolac tablets	P.O.	200 mg	Administration began before ENR	Continued through 52W	Complication
al-so-w03	Limaprost alfadex tablets	P.O.	10 µg	Administration began before ENR	Continued through 52W	Complication

ENR – enrollment, AE - adverse event, LE – left eye.

Concomitant drugs *(continued)*

Subject ID Code	Drug	Administration Route	Dose	Administration Start Date	Administration End Date	Reason for Administration
al-so-w03	Hachimijiogan	P.O.	7.5 g	Administration began before ENR	Continued through 52W	Complication
al-so-w03	Dorzolamide hydrochloride	Instillation (RE only)	3 administrations	October 04, 2017	June 19, 2018	Treatment of AE
al-so-w03	Latanoprost	Instillation RE only)	1 administration	October 25, 2017	November 13, 2017	Treatment of AE
al-so-w03	Influenza HA vaccine	Other (S.C.)	Sufficient quantity	November 02, 2017	November 02, 2017	Prophylactic administration (influenza prevention)
al-so-w03	Latanoprost	Instillation (RE only)	1 administration	February 21, 2018	Continued through 52W	Treatment of AE
al-so-w03	Gatifloxacin hydrate	Instillation (RE only)	4 administrations	July 24, 2017	Continued through 52W	Treatment related to this study
al-so-w03	Betamethasone sodium phosphate	Instillation (RE only)	4 administrations	July 24, 2017	May 30, 2018	Treatment related to this study
al-so-w03	Acetaminophen	P.O.	400 mg	November 20, 2017	November 20, 2017	Prophylactic administration (pain after intravitreal injection)
al-so-w03	Fluorometholone	Instillation (RE only)	4 administrations	May 30, 2018	Continued through 52W	Treatment related to this study
al-so-w03	Acetaminophen	P.O.	400 mg	June 14, 2018	June 14, 2018	Prophylactic administration (pain after intravitreal injection)

ENR – enrollment, AE - adverse event, RE – right eye.

Concomitant drugs *(continued)*

Subject ID Code	Drug	Administration Route	Dose	Administration Start Date	Administration End Date	Reason for Administration
al-so-w03	Brinzolamide	Instillation (RE only)	2 administrations	June 20, 2018	Continued through 52W	Treatment of AE
al-so-w04	Olmesartan medoxomil	P.O.	20 mg	Administration began before ENR	Continued through 52W	Complication
al-so-w04	Atorvastatin calcium hydrate	P.O.	10 mg	Administration began before ENR	Continued through 52W	Complication
al-so-w04	Zolpidem tartrate	P.O.	5 mg	Administration began before ENR	Continued through 52W	Complication
al-so-w04	Vonoprazan fumarate	P.O.	20 mg	Administration began before ENR	Continued through 5W	Complication
al-so-w04	Magnesium oxide	P.O.	250 mg	Administration began before ENR	Continued through 52W	Complication
al-so-w04	Latanoprost	Instillation (RE only)	1 administration	August 16, 2017	September 11, 2017	Complication
al-so-w04	Moxifloxacin hydrochloride	Instillation (RE only)	4 administrations	September 26, 2017	Continued through 52W	Treatment related to this study
al-so-w04	Betamethasone sodium phosphate	Instillation (RE only)	4 administrations	September 13, 2017	May 23, 2018	Treatment related to this study
al-so-w04	Cefcapene pivoxil hydrochloride hydrate	P.O.	300 mg	September 13, 2017	September 15, 2017	Prophylactic administration (postoperative infection)

ENR – enrollment, AE - adverse event, RE – right eye.

Concomitant drugs *(continued)*

Subject ID Code	Drug	Administration Route	Dose	Administration Start Date	Administration End Date	Reason for Administration
al-so-w04	Gatifloxacin hydrate	Instillation (RE only)	4 administrations	September 13, 2017	September 25, 2017	Treatment related to this study
al-so-w04	VONOSAP Pack 400	P.O.	2 tablets	November 06, 2017	November 12, 2017	Complication
al-so-w04	Miya BM fine granules	P.O.	80 mg	November 06, 2017	November 12, 2017	Complication
al-so-w04	Azelnidipine	P.O.	16 mg	Administration began before ENR	Continued through 52W	Complication
al-so-w04	Brinzolamide	Instillation (RE only)	2 administrations	October 23, 2017	July 11, 2018	Treatment of AE
al-so-w04	Latanoprost	Instillation (RE only)	1 administration	October 16, 2017	Continued through 52W	Treatment of AE
al-so-w04	Aiphagan ophthalmic solution	Instillation (RE only)	2 administrations	May 23, 2018	Continued through 52W	Treatment of AE
al-so-w04	Betamethasone sodium phosphate	Instillation (RE only)	2 administrations	May 23, 2018	Continued through 52W	Treatment of AE
al-so-w04	Fluorometholone solution 0.1%	Instillation (RE only)	4 administrations	June 02, 2018	Continued through 52W	Treatment of underlying disease
al-so-w04	COSOPT ophthalmic solution	Instillation (RE only)	2 administrations	July 12, 2018	Continued through 52W	Treatment of AE

AE - adverse event, RE – right eye.

Concomitant drugs *(continued)*

Subject ID Code	Drug	Administration Route	Dose	Administration Start Date	Administration End Date	Reason for Administration
OSK-al-so-w-01	Cefcapene pivoxil hydrochloride	P.O.	300 mg	September 22, 2017	September 25, 2017	Prophylactic administration (infection prevention)
OSK-al-so-w-01	Teprenone	P.O.	150 mg	September 22, 2017	September 25, 2017	Prophylactic administration (gastritis prevention)
OSK-al-so-w-01	Moxifloxacin hydrochloride	Instillation (RE only)	3 drops	September 22, 2017	December 15, 2017	Prophylactic administration (infection prevention)
OSK-al-so-w-01	Betamethasone sodium phosphate	Instillation (RE only)	3 drops	September 22, 2017	September 22, 2017	Treatment of AE
OSK-al-so-w-01	Betamethasone sodium phosphate	Instillation (RE only)	15 drops	September 23, 2017	September 25, 2017	Treatment of AE
OSK-al-so-w-01	Betamethasone sodium phosphate	Instillation (RE only)	4 drops	September 26, 2017	October 24, 2017	Treatment of AE
OSK-al-so-w-01	Methylprednisolone sodium succinate	I.V.	1000 mg	September 27, 2017	September 29, 2017	Prophylactic administration (rejection prevention)
OSK-al-so-w-01	Sodium rabeprazole	P.O.	10 mg	September 27, 2017	September 29, 2017	Prophylactic administration (gastritis prevention)
OSK-al-so-w-01	Esomeprazole magnesium hydrate	P.O.	20 mg	September 30, 2017	October 27, 2017	Prophylactic administration (gastritis prevention)
OSK-al-so-w-01	Prednisolone	P.O.	20 mg	September 30, 2017	October 06, 2017	Prophylactic administration (rejection prevention)
OSK-al-so-w-01	Prednisolone	P.O.	15 mg	October 07, 2017	October 13, 2017	Prophylactic administration (rejection prevention)

AE - adverse event, RE – right eye.

Concomitant drugs *(continued)*

Subject ID Code	Drug	Administration Route	Dose	Administration Start Date	Administration End Date	Reason for Administration
OSK-al-so-w-01	Prednisolone	P.O.	10 mg	October 14, 2017	October 20, 2017	Prophylactic administration (rejection prevention)
OSK-al-so-w-01	Prednisolone	P.O.	5 mg	October 21, 2017	October 27, 2017	Prophylactic administration (rejection prevention)
OSK-al-so-w-01	Alendronate sodium hydrate (weekly)	P.O.	35 mg	September 30, 2017	October 21, 2017	Prophylactic administration (osteoporosis prevention)
OSK-al-so-w-01	Betamethasone sodium phosphate	Instillation (RE only)	4 drops	October 25, 2017	December 15, 2017	Prophylactic administration (inflammation prevention)

RE – right eye.

Table S14. Concomitant therapies in all cases.

Subject ID Code	Concomitant Therapy	Start Date	End Date	Reason for Therapy
al-so-w01	Eylea intravitreal injection, RE	May 08, 2017	May 08, 2017	Treatment of underlying disease
al-so-w01	sub-Tenon conjunctival injection of triamcinolone, RE	May 08, 2017	May 08, 2017	Treatment of AE
al-so-w01	sub-Tenon conjunctival injection of triamcinolone, RE	June 12, 2017	June 12, 2017	Treatment of AE
al-so-w01	sub-Tenon conjunctival injection of triamcinolone, RE	August 21, 2017	August 21, 2017	Treatment of AE
al-so-w01	Eylea intravitreal injection, RE	October 16, 2017	October 16, 2017	Treatment of underlying disease
al-so-w01	Eylea intravitreal injection, RE	December 13, 2017	December 13, 2017	Treatment of underlying disease
al-so-w01	Eylea intravitreal injection, LE	January 17, 2018	January 17, 2018	Complication
al-so-w01	Eylea intravitreal injection, LE	March 07, 2018	March 07, 2018	Complication
al-so-w01	Eylea intravitreal injection, LE	June 19, 2017	June 19, 2017	Complication
al-so-w02	Eylea intravitreal injection, LE	November 29, 2017	November 29, 2017	Treatment of underlying disease
al-so-w02	Vitrectomy, LE	January 15, 2018	January 15, 2018	Treatment of AE
al-so-w02	Eylea intravitreal injection, LE	September 20, 2017	September 20, 2017	Treatment of underlying disease
al-so-w02	sub-Tenon conjunctival injection of triamcinolone, LE	October 16, 2017	October 16, 2017	Treatment of AE
al-so-w02	Eylea intravitreal injection, LE	January 15, 2018	January 15, 2018	Treatment of underlying disease
al-so-w03	Eylea intravitreal injection, RE	November 20, 2017	November 20, 2017	Treatment of underlying disease
al-so-w03	Eylea intravitreal injection, RE	June 14, 2018	June 14, 2018	Treatment of underlying disease
al-so-w04	Eylea intravitreal injection, LE	September 19, 2017	September 19, 2017	Complication
al-so-w04	Eylea intravitreal injection, LE	December 13, 2017	December 13, 2017	Complication
al-so-w04	Eylea intravitreal injection, LE	March 06, 2018	March 06, 2018	Complication
al-so-w04	Eylea intravitreal injection, RE	August 16, 2018	August 16, 2018	Treatment of underlying disease

AE - adverse event, RE – right eye, LE – left eye.

Table S15. Lists of adverse events in all cases.

Subject ID Code	Adverse Event (AE)	Serious/Non-serious	Date Occurred	Severity	Treated	Outcome	Causality	Continued Participation in Study	AE Associated with Transplantation Surgery	AE Caused by iPS Cell-derived RPE Cells
al-so-w01	Corneal epithelial detachment	Non-serious	March 28, 2017	Grade 2 (moderate)	Yes	Resolved (April 11, 2017)	Related (directly, transplantation surgery procedure)	Continued	Other	N/A
al-so-w01	Increased postoperative inflammation	Non-serious	March 30, 2017	Grade 2 (moderate)	No	Resolved (April 4, 2017)	Related (directly, transplantation surgery procedure)	Continued	Other	N/A
al-so-w01	Allergic reaction	Non-serious	April 24, 2017	Grade 2 (moderate)	Yes	Resolved (April 25, 2017)	Not related (contrast media allergy; caused by patient predisposition)	Continued	N/A	N/A
al-so-w01	Suspected mild rejection	Non-serious	April 24, 2017	Grade 2 (moderate)	Yes	Resolved (December 4, 2017)	Related (directly, transplanted RPE cells)	Continued	N/A	Other
al-so-w01	Allergic reaction	Non-serious	June 26, 2017	Grade 2 (moderate)	Yes	Resolved (June 27, 2017)	Not related (contrast media allergy; caused by patient predisposition)	Continued	N/A	N/A

Adverse Events (continued)

Subject ID Code	Adverse Event (AE)	Serious/Non-serious	Date Occurred	Severity	Treated	Outcome	Causality	Continued Participation in Study	AE Associated with Transplantation Surgery	AE Caused by iPS Cell-derived RPE Cells
al-so-w01	Allergic reaction	Non-serious	September 11, 2017	Grade 2 (moderate)	Yes	Resolved (September 12, 2017)	Not related (contrast media allergy; caused by patient predisposition)	Continued	N/A	N/A
al-so-w01	Allergic reaction	Non-serious	November 4, 2017	Grade 2 (moderate)	Yes	Resolved (November 8, 2017)	Not related (caused by patient predisposition)	Continued	N/A	N/A
al-so-w01	Allergic reaction	Non-serious	March 14, 2018	Grade 2 (moderate)	Yes	Resolved (March 14, 2018)	Not related (contrast media allergy, caused by patient predisposition)	Continued	N/A	N/A
al-so-w01	Subretinal hemorrhage	Non-serious	March 14, 2018	Grade 1 (Mild)	No	Remitted (September 18, 2018)	Not related (worsening of underlying disease)	Continued	N/A	N/A
al-so-w02	Increased intraocular pressure, left	Non-serious	August 10, 2017	Grade 2 (moderate)	Yes	No change (July 5, 2018)	Related (weak relationship, medical intervention other than that described above)	Continued	N/A	N/A

Adverse Events (continued)

Subject ID Code	Adverse Event (AE)	Serious/Non-serious	Date Occurred	Severity	Treated	Outcome	Causality	Continued Participation in Study	AE Associated with Transplantation Surgery	AE Caused by iPS Cell-derived RPE Cells
al-so-w02	Mild rejection	Non-serious	October 4, 2017	Grade 2 (moderate)	Yes	Resolved (November 13, 2017)	Related (directly, transplanted RPE cells)	Continued	N/A	Other
al-so-w02	Retinal edema associated with epiretinal membrane	Serious	October 27, 2017	Grade 2 (moderate)	Yes	Resolved (January 15, 2018)	Related (strong relationship, transplantation surgery procedure)	Continued	Other	Other
al-so-w02	Neurally mediated syncope	Non-serious	December 15, 2017	Grade 1 (Mild)	No	Resolved (December 15, 2017)	Not related [due to patient predisposition or concomitant drug (alpha-blocker)]	Continued	N/A	N/A
al-so-w02	Cystoid macular edema	Non-serious	March 28, 2018	Grade 1 (Mild)	No	No change (July 9, 2018)	Not related (worsening of underlying disease)	Continued	N/A	N/A
al-so-w03	Increased intraocular pressure, right	Non-serious	October 4, 2017	Grade 1 (Mild)	Yes	-	Related (weak relationship, medical intervention other than that described above)	Continued	N/A	N/A

Adverse Events (continued)

Subject ID Code	Adverse Event (AE)	Serious/Non-serious	Date Occurred	Severity	Treated	Outcome	Causality	Continued Participation in Study	AE Associated with Transplantation Surgery	AE Caused by iPS Cell-derived RPE Cells
al-so-w03	Hyperglycemia	Non-serious	August 21, 2017	Grade 1 (Mild)	No	Resolved (October 16, 2017)	Not related (because increase was transient and considered to be in the range of physiological change)	Continued	N/A	N/A
al-so-w04	Cystoid macular edema	Non-serious	October 30, 2017	Grade 1 (Mild)	No	Remitted (September 12, 2018)	Not related (worsening of underlying disease)	Continued	N/A	N/A
al-so-w04	Superficial punctate keratopathy	Non-serious	November 6, 2017	Grade 1 (Mild)	No	Remitted (September 12, 2018)	Not related [effect of concomitant drug (hypotensive drug)]	Continued	N/A	N/A
al-so-w04	Macular hole (Macular pseudohole)	Non-serious	May 23, 2018	Grade 2 (moderate)	Yes	No change (September 12, 2018)	Not related (due to underlying disease)	Continued	N/A	N/A
al-so-w04	Increased intraocular pressure	Non-serious	October 16, 2017	Grade 2 (moderate)	Yes	Remitted (September 12, 2018)	Related (strong relationship; in addition: effect of concomitant medication and glaucoma, a complication)	Continued	N/A	N/A

Adverse Events (continued)

Subject ID Code	Adverse Event (AE)	Serious/Non-serious	Date Occurred	Severity	Treated	Outcome	Causality	Continued Participation in Study	AE Associated with Transplantation Surgery	AE Caused by iPS Cell-derived RPE Cells
OSK-al-so-w-01	Aseptic endophthalmitis	Serious	September 22, 2017	Grade 2 (moderate)	Yes	Resolved (October 24, 2017)	Related (weak relationship, transplantation surgery procedure)	Continued	Other	N/A

al-so-w01 is Case 1, al-so-w02 is Case 2, al-so-w03 is Case 3, al-so-w04 is Case 4, and OSK-al-so-w01 is Case 5. Other in AE associated with transplantation surgery indicates the adverse events with exception of retinal/choroidal/vitreous hemorrhage or retinal detachment caused by transplantation surgery. Other in AE caused by iPSC-derived RPE cells indicates the adverse events with exception of poor engraftment of transplanted cells, immune rejection, excessive cell proliferation, and tumorigenicity (Suspected immune rejection is “other”). N/A – Not Applicable.

Table S16. Inflammatory factors for primers and probe in qRT-PCR.

No.	Molecule	Left primer	Right primer	Probe*
1	Actin, beta (β-actin)	ccaaccgcgagaagatga	ccagaggcgtacagggatag	#64
2	Pigment epithelium derived factor (PEDF)	gtgtggagctgcagcgtat	tccaatgcagaggagtagca	#57
3	Transforming growth factor, beta 2 (TGFβ2)	ccaaagggtacaatgccaac	cagatgcttctggatttatggtatt	#67
4	CD3e molecule, epsilon (CD3ε)	caaggccaagcctgtgac	tcatagtctgggttgggaaca	#49
5	Allograft inflammatory factor 1 (AIF1/Iba1)	ccaaaccagggatttacagg	cgtctaggaattgcttgttgatct	#4
6	Interferon, gamma (IFN-γ)	ggcattttgaagaattggaaag	tttggatgctctggtcatctt	#21

In quantitative RT-PCR, we extracted RNA from the epiretinal membrane tissue in Case 2, cultured iPS-RPE cells, human primary RPE cells, and peripheral blood cells. *Probe - The probe in the Roche Universal Probe Library was used for our qRT-PCR assay.

Supplementary References

1. Wang, K.; Li, M.; Hadley, D.; Liu, R.; Glessner, J.; Grant, S. F.; Hakonarson, H.; Bucan, M., PennCNV: an integrated hidden Markov model designed for high-resolution copy number variation detection in whole-genome SNP genotyping data. *Genome research* **2007,** 17, (11), 1665-74.
2. Gonzalez, J. R.; Rodriguez-Santiago, B.; Caceres, A.; Pique-Regi, R.; Rothman, N.; Chanock, S. J.; Armengol, L.; Perez-Jurado, L. A., A fast and accurate method to detect allelic genomic imbalances underlying mosaic rearrangements using SNP array data. *BMC bioinformatics* **2011,** 12, 166.
3. Koboldt, D. C.; Zhang, Q.; Larson, D. E.; Shen, D.; McLellan, M. D.; Lin, L.; Miller, C. A.; Mardis, E. R.; Ding, L.; Wilson, R. K., VarScan 2: somatic mutation and copy number alteration discovery in cancer by exome sequencing. *Genome Res* **2012,** 22, (3), 568-76.
4. Otsu, N., A Tlreshold Selection Method from Gray-Level Histograms. *IEEE Trans. Systems, Man and Cybernetics* **1979,** 9, 62-66.
5. Rausch, T.; Zichner, T.; Schlattl, A.; Stutz, A. M.; Benes, V.; Korbel, J. O., DELLY: structural variant discovery by integrated paired-end and split-read analysis. *Bioinformatics* **2012,** 28, (18), i333-i339.
6. Teschendorff, A. E.; Marabita, F.; Lechner, M.; Bartlett, T.; Tegner, J.; Gomez-Cabrero, D.; Beck, S., A beta-mixture quantile normalization method for correcting probe design bias in Illumina Infinium 450 k DNA methylation data. *Bioinformatics* **2013,** 29, (2), 189-96.
7. Kim, J. G.; Takeshima, H.; Niwa, T.; Rehnberg, E.; Shigematsu, Y.; Yoda, Y.; Yamashita, S.; Kushima, R.; Maekita, T.; Ichinose, M.; Katai, H.; Park, W. S.; Hong, Y. S.; Park, C. H.; Ushijima, T., Comprehensive DNA methylation and extensive mutation analyses reveal an association between the CpG island methylator phenotype and oncogenic mutations in gastric cancers. *Cancer Lett* **2013,** 330, (1), 33-40.
8. Mandai, M.; Watanabe, A.; Kurimoto, Y.; Hirami, Y.; Morinaga, C.; Daimon, T.; Fujihara, M.; Akimaru, H.; Sakai, N.; Shibata, Y.; Terada, M.; Nomiya, Y.; Tanishima, S.; Nakamura, M.; Kamao, H.; Sugita, S.; Onishi, A.; Ito, T.; Fujita, K.; Kawamata, S.; Go, M. J.; Shinohara, C.; Hata, K. I.; Sawada, M.; Yamamoto, M.; Ohta, S.; Ohara, Y.; Yoshida, K.; Kuwahara, J.; Kitano, Y.; Amano, N.; Umekage, M.; Kitaoka, F.; Tanaka, A.; Okada, C.; Takasu, N.; Ogawa, S.; Yamanaka, S.; Takahashi, M., Autologous Induced Stem-Cell-Derived Retinal Cells for Macular Degeneration. *N Engl J Med* **2017,** 376, (11), 1038-1046.
9. Sugita, S.; Ogawa, M.; Shimizu, N.; Morio, T.; Ohguro, N.; Nakai, K.; Maruyama, K.; Nagata, K.; Takeda, A.; Usui, Y.; Sonoda, K. H.; Takeuchi, M.; Mochizuki, M., Use of a comprehensive polymerase chain reaction system for diagnosis of ocular infectious diseases. *Ophthalmology* **2013,** 120, (9), 1761-8.
10. Sugita, S.; Shimizu, N.; Watanabe, K.; Mizukami, M.; Morio, T.; Sugamoto, Y.; Mochizuki, M., Use of multiplex PCR and real-time PCR to detect human herpes virus genome in ocular fluids of patients with uveitis. *The British journal of ophthalmology* **2008,** 92, (7), 928-32.
11. Sugita, S.; Iwasaki, Y.; Makabe, K.; Kimura, T.; Futagami, T.; Suegami, S.; Takahashi, M., Lack of T Cell Response to iPSC-Derived Retinal Pigment Epithelial Cells from HLA Homozygous Donors. *Stem Cell Reports* **2016,** 7, (4), 619-634.
12. Cense, B.; Chen, T. C.; Park, B. H.; Pierce, M. C.; de Boer, J. F., Invivo depth-resolved birefringence measurements of the human retinal nerve fiber layer by polarization-sensitive optical coherence tomography. *Optics letters* **2002,** 27, (18), 1610-2.
13. Gotzinger, E.; Pircher, M.; Hitzenberger, C. K., High speed spectral domain polarization sensitive optical coherence tomography of the human retina. *Optics express* **2005,** 13, (25), 10217-29.
14. Yamanari, M.; Lim, Y.; Makita, S.; Yasuno, Y., Visualization of phase retardation of deep posterior eye by polarization-sensitive swept-source optical coherence tomography with 1-microm probe. *Optics express* **2009,** 17, (15), 12385-96.
15. Pircher, M.; Gotzinger, E.; Findl, O.; Michels, S.; Geitzenauer, W.; Leydolt, C.; Schmidt-Erfurth, U.; Hitzenberger, C. K., Human macula investigated in vivo with polarization-sensitive optical coherence tomography. *Invest Ophthalmol Vis Sci* **2006,** 47, (12), 5487-94.
16. Baumann, B.; Baumann, S. O.; Konegger, T.; Pircher, M.; Gotzinger, E.; Schlanitz, F.; Schutze, C.; Sattmann, H.; Litschauer, M.; Schmidt-Erfurth, U.; Hitzenberger, C. K., Polarization sensitive optical coherence tomography of melanin provides intrinsic contrast based on depolarization. *Biomedical optics express* **2012,** 3, (7), 1670-83.
17. Gotzinger, E.; Pircher, M.; Geitzenauer, W.; Ahlers, C.; Baumann, B.; Michels, S.; Schmidt-Erfurth, U.; Hitzenberger, C. K., Retinal pigment epithelium segmentation by polarization sensitive optical coherence tomography. *Optics express* **2008,** 16, (21), 16410-22.
18. Yamanari, M.; Tsuda, S.; Kokubun, T.; Shiga, Y.; Omodaka, K.; Aizawa, N.; Yokoyama, Y.; Himori, N.; Kunimatsu-Sanuki, S.; Maruyama, K.; Kunikata, H.; Nakazawa, T., Estimation of Jones matrix, birefringence and entropy using Cloude-Pottier decomposition in polarization-sensitive optical coherence tomography. *Biomedical optics express* **2016,** 7, (9), 3551-3573.
19. Yamanari, M.; Tsuda, S.; Kokubun, T.; Shiga, Y.; Omodaka, K.; Yokoyama, Y.; Himori, N.; Ryu, M.; Kunimatsu-Sanuki, S.; Takahashi, H.; Maruyama, K.; Kunikata, H.; Nakazawa, T., Fiber-based polarization-sensitive OCT for birefringence imaging of the anterior eye segment. *Biomedical optics express* **2015,** 6, (2), 369-89.
